# Harnessing Dual Power: Genistein-Loaded Pumpkisomes in Pullulan Microneedles for Potent Antioxidant and Anticancer Therapy Against Ehrlich Ascites Carcinoma and Breast Cancer Cells

**DOI:** 10.3390/pharmaceutics18010036

**Published:** 2025-12-26

**Authors:** Sammar Fathy Elhabal, Mai S. Shoela, Mohamed Fathi Mohamed Elrefai, Fatma E. Hassan, Suzan Awad AbdelGhany Morsy, Wedian Younis Abdelgawad, Sahar K. Ali, Passant M. Mohie, Amal M. Elsharkawy, Tassneim M. Ewedah, Ibrahim S. Mousa, Marwa A. Fouad, Shady Allam, Ahmed Mohsen Elsaid Hamdan

**Affiliations:** 1 Department of Pharmaceutics and Industrial Pharmacy, Faculty of Pharmacy, Modern University for Technology and Information (MTI), Mokattam, Cairo 11571, Egypt; 2 Department of Clinical Pharmacology, Faculty of Medicine, Alexandria University, Alexandria 21526, Egypt; mai.shoala@alexmed.edu.eg (M.S.S.); susan.ulghani@alexmed.edu.eg (S.A.A.M.); bassant.muhyeldin@alexmed.edu.eg (P.M.M.); amal.elsharkawy@alexmed.edu.eg (A.M.E.); 3 Department of Anatomy and Embryology, Faculty of Medicine, Ain Shams University, Cairo 11591, Egypt; mohamedfathianatomy@yahoo.com; 4 Department of Basic Medical Sciences, Faculty of Medicine, Aqaba Medical Sciences University, Aqaba 77110, Jordan; 5 Medical Physiology Department, Faculty of Medicine, Cairo University, Kasr Alainy, Giza 11562, Egypt; fatma.e.elsayed@kasralainy.edu.eg; 6 Department of Physiology, General Medicine Practice Program, Batterjee Medical College, Jeddah 21442, Saudi Arabia; 7 Pathological Sciences Department, MBBS Program, Fakeeh College for Medical Sciences, Jeddah 21461, Saudi Arabia; 8 Department of Pharmaceutics, Egyptian Drug Authority (Formerly National Organization for Drug Control and Research “NODCAR”), Giza 15301, Egypt; wedianyounis@yahoo.com; 9 Department of Clinical Pharmacology, Faculty of Medicine, Zagazig University, Zagazig 44519, Egypt; skaly@medicine.zu.edu.eg; 10 Pharmaceutics and Pharmaceutical Technology Department, Faculty of Pharmacy, Egyptian Russian University, Cairo 11829, Egypt; tassneim-mohammed@eru.edu.eg; 11 Pharmaceutics Department, Faculty of Pharmacy, Sinai University, Al-Arish 45511, Egypt; ibrahim.salah@su.edu.eg; 12 Department of Pharmaceutics and Pharmaceutical Technology, Faculty of Pharmacy, Deraya University, Minia 61768, Egypt; marwa.fouad@deraya.edu.eg; 13 Department of Pharmacology and Toxicology, Faculty of Pharmacy, Menoufia University, Menoufia 32811, Egypt; shady_allam@phrm.menofia.edu.eg; 14 Department of Pharmacology and Toxicology, Faculty of Pharmacy, Menoufia National University, Cairo-Alexandria Agricultural Road, Menoufia 32511, Egypt; 15 Department of Pharmacology and Toxicology, Faculty of Pharmacy, University of Tabuk, Tabuk 71491, Saudi Arabia; 16 Prince Fahad bin Sultan Chair for Biomedical Research (PFSCBR), Tabuk 74191, Saudi Arabia

**Keywords:** breast cancer, EGFR, Ehrlich ascites carcinoma, genistein, microneedles, nanovesicles, natural products, oxidative stress, pullulan, pumpkin seed oil

## Abstract

**Background/Objectives:** Breast cancer remains one of the leading causes of cancer-related mortality. Still, limited drug delivery systems for genistein, a powerful natural anticancer agent, draw significant attention. We aimed to develop a co-therapeutic/synergistic dual-compartment system; genistein-loaded pumpkisome nanovesicles (GNS-PKs) incorporated into pullulan microneedle patches (MNs), and to explore its anticancer activity. **Methods:** GNS-PKs were prepared and characterized for particle size (P.S), polydispersity (PDI), zeta potential (Z.P), encapsulation efficiency (E.E%), and stability. Afterward, they were embedded in pullulan-dissolving microneedle arrays and characterized for release kinetics, mechanical strength, and in vitro cytotoxicity. The in vivo efficacy was evaluated in mice with solid Ehrlich Ascites Carcinoma (EAC), focusing on tumor volume, oxidative stress, inflammatory cytokines, Epidermal Growth Factor (EGFR) expression biomarkers, and histopathological analysis. **Results:** The optimized nanovesicles had a particle size of 170 nm, a zeta potential of −42 mV, and an entrapment efficiency of up to 92%. Pullulan microneedles demonstrated significantly high mechanical strength and effective deep penetration. In addition to, it markedly decreased MCF-7 cellular viability (IC_50_ = 3.5 µg/mL). Besides, it had a 76% reduction in tumor volume, significantly increased the antioxidant activity (SOD, CAT, GSH), decreased the levels of inflammatory biomarkers (IL-6, COX-2, NF-κB), and markedly downregulated the EGFR expression (*p* < 0.0001). Histological study revealed decreased mitotic activity and large tumor cells, with minimal systemic damage. **Conclusions:** GNS-PKs-pullulan microneedle system offers a hope for an innovative, potent, effective, and non-invasive strategy for breast cancer treatment with high antitumor efficacy.

## 1. Introduction

Breast cancer (BC) is the world’s most often diagnosed malignancy and the leading cause of cancer-related death in women [1]. Breast carcinoma is the second most reported type of cancer after lung cancer [2]. It is one of the deadliest diseases, accounting for 15% of cancer-related deaths in women and 23% of cancer diagnoses [3]. Amplification of human epidermal growth factor receptor 2 (HER2) has been found in roughly 15–25% of breast cancer cases, as well as in other neoplasms such as gastric/gastroesophageal, colon, lung, and bladder cancers [4,5]. Notably, HER2 amplification is associated with the worst outcome in breast cancer patients. Despite the availability of anti-HER2 therapies, a substantial number of patients encounter tumor recurrence or metastasis within one year of starting treatment, underscoring the urgent need for the discovery of new therapeutic alternatives [6,7]. HER2-targeted monoclonal antibodies and tyrosine kinase inhibitors have significantly altered clinical management; however, their prolonged use is constrained by issues such as drug resistance, cardiotoxicity, elevated systemic exposure, and insufficiently sustained intratumoral drug concentrations. The identified limitations highlight the need for alternative strategies that can effectively deliver bioactive compounds locally and safely while reducing systemic toxicity. Despite advancements in early detection and targeted therapies, therapeutic resistance and disease progression continue to pose substantial clinical challenges [8,9]. Contemporary cancer treatment guidelines include surgery, radiotherapy, chemotherapy, immunotherapy, cancer vaccines, photodynamic therapy, stem cell transplantation, and combinations. Meanwhile, the limited metastasis, toxicity, nonspecificity, and poor absorptivity are the key drawbacks that hinder the effective and successful treatment of breast cancer [10,11]. Type, stage, and site determine the strategies of cancer treatment [12,13]. Cytotoxic and cytostatic chemotherapy can be used alongside other cancer treatments. Cardiovascular, gastrointestinal, hematologic, pulmonary, diarrhea, sensory neuropathy, and neutropenia can result from carboplatin, cisplatin, oxaliplatin, and melphalan [14,15]. These compounds treat numerous tumors but are costly, complicated, toxic, and environmentally hazardous. The current therapeutic limitations, resistance to HER2-directed agents, systemic toxicity of cytotoxic drugs, and inadequate local drug exposure, highlight a significant unmet need for minimally invasive delivery systems with maintained constant drug concentrations at the tumor site.

There are great attention of phytochemicals and their anticancer activities [1,16]. They antagonize cancer cell-activating proteins, enzymes, and signaling systems, stimulate DNA repair, enhance protective enzyme synthesis, and activate antioxidant activity [17,18]. Lignans, flavonoids, alkaloids, vitamins, terpenes, taxanes, saponins, minerals, oils, gums, glycosides, and biological substances are metabolites with strong anticancer properties [19,20]. Plant-derived bioactive phytochemicals such as curcumin, resveratrol, and fisetin; flavonoids such as genistein and baicalein; terpenoids such as β-elemene and carnosol; and bioactive oils and alkaloids from *Salvadora persica* and *Cucurbita pepo*, can effectively mitigate breast cancer across different subtypes [21,22]. These medicines prevent tumor genesis, growth, and metastasis, and promote apoptosis and cell cycle arrest via multiple overlapping biological pathways [23,24]. The pharmacological mechanisms of action of those anticancer drugs include reducing oxidative stress, inhibiting angiogenesis, and boosting detoxification and immunity, demonstrating their biological flexibility [25,26]. The clinical and therapeutic path from lab to bedside for these natural compounds is challenging. Few well-designed human clinical trials exist because of the high risk posed by their cytotoxicity. Dietary phytochemicals and functional plant extracts support global breast cancer prevention and treatment trends toward safer, less toxic, and more holistic methods [27,28]. Fisetin, genistein, pumpkin seed oil, *Nigella sativa* oil, and *Salvadora persica* extracts have therapeutic potential and are underexplored for their anticancer properties, unlike curcumin or resveratrol [1,18,29]. Molecular pharmacology, nutritional science, and natural product chemistry must identify, refine, and validate medicinal molecules.

Genistein (GNS), 4′,5,7-trihydroxyisoflavone, is a natural isoflavone compound that can be found in soybeans in relatively high concentrations (up to 3 mg/g) [30,31]. It has attracted significant recent attention due to its structural similarity to 17β-estradiol and its estrogenic characteristics [32,33]. GNS has been recognized as a potential therapeutic agent for breast, lung, and colon cancers due to its significant antioxidant, anti-inflammatory, and estrogenic properties [21,34]. In 2014, the American Institute for Cancer Research and the World Cancer Research Fund suggested that, despite limited research, there is sufficient evidence to propose that isoflavones, particularly GNS, may improve breast cancer survival rates [35,36]. Investigations on GNS have yielded ambiguous results, indicating its potential to enhance cancer proliferation in specific cellular and animal models. The prolonged effects of GNS on breast cancer continue to be a contentious issue [37]. GNS demonstrates estrogenic activity, inhibits angiogenesis, promotes apoptosis and autophagy, and regulates immune responses and cell cycle regulation [38]. The emerging evidence that GNS therapeutic impact differs by patient demographics, particularly among pre-pubescent, menopausal, and postmenopausal women, emphasizes the need for tailored treatment approaches [39]. GNS has poor pharmacokinetics, low water solubility, and rapid metabolism, resulting in minimal bioavailability despite its therapeutic promise. Nanotechnology-based formulations, including lipid and nanoparticle formulations, improve the bioavailability, stability, and therapeutic efficacy of GNS [40]. In order to enhance the solubility and the bioavailability of GNS, a variety of drug delivery strategies have been used, including self-nanoemulsified systems, chitosan-based nanoparticles, and lipid nanoparticles. However, the potential for cutaneous delivery is limited. The stratum corneum, the skin’s outermost layer, is high in lipids and proteins and regulates modest absorption rates, which lipid-based delivery systems can help enhance. Lipid nanoparticles have been shown to promote medication penetration through the skin. This relates to the creation of an occlusive layer that blocks water loss from the skin [18,41]. Furthermore, these nanoparticles improve skin hydration and exhibit adhesive properties, facilitating efficient drug delivery to the desired area within the epidermis or dermis [12]. Solid lipid nanoparticles and nanostructured lipid carriers (NLCs) are highly explored lipid nanoparticles due to their ease of manufacture, low cost, and stability. GNS encapsulated alginate-chitosan nanoparticles were incorporated into dissolvable microneedle arrays of Povidone K90/PVA biodegradable matrix, which exhibited controlled release (75 ± 1.24%) for 144 h with elevated drug content (97.00 ± 0.42%) [42]. Functionalized nanovesicles and Microneedles (MNs) make focused medication delivery, reduce off-target toxicity, and prolong tumor release more effectively [43,44]. Our study aims to explore the therapeutic efficacy of advanced nanoformulations for GNS, thereby enhancing the significance of overcoming standard cancer therapies and improving patient compliance by evaluating the therapeutic potential of an innovative drug-delivery method for GNS.

Pumpkin seed oil (P.O.) contains phytosterols, lignans, antioxidative phenolic compounds, and unsaturated fatty acids, with oleic (43.8%) and linoleic (33.1%) acids being the most prevalent [45]. Oleic acid (OA) and linoleic acid (LA) have been shown to improve cancer treatment in multiple studies [46]. This study defines the ‘dual-therapeutic system’ as comprising (i) genistein, the principal flavonoid anticancer agent, and (ii) unsaturated fatty acids derived from pumpkin oil, specifically oleic acid and linoleic acid, both of which demonstrate antiproliferative and HER2-modulatory effects. Consequently, pumpkisomes serve dual roles as nanocarriers and bioactive therapeutic agents. The proposed mechanisms are: (a) GNS-mediated suppression of tyrosine kinase/estrogen-receptor pathways, (b) OA-induced HER2 downregulation and apoptosis, and (c) LA-mediated fatty-acid-synthase inhibition and cell-cycle arrest [47]. LA inhibits fatty acid synthase in breast cancer cells, leading to apoptosis, invasion, metastasis, and cell cycle arrest [47]. It has been showed that OA and LA can modulate the chemosensitivity of MDA-MB-231 cells during paclitaxel-based therapy [48]. Gebicki and Hicks proved that unsaturated fatty acids could form closed lipid bilayer membrane vesicles, known as ufasomes [49]. Ufasomes are more dynamic than double-chain amphiphiles and micelles due to their single-chain structure [50]. They have several advantages over phospholipid vesicles, including greater stability, increased cell membrane fluidity, and disruption of tight junctions [50,51]. The combination of genistein and P.O.-based nanovesicles is mechanistically synergistic because oleic and linoleic acids modulate HER2 overexpression, inhibit fatty acid synthase, and increase breast cancer cell susceptibility to apoptosis. In contrast, genistein suppresses tyrosine kinase signaling, modulates estrogen receptor pathways, and enhances antioxidant defense. Convergent nodes at EGFR/HER2, NF-κB, and oxidative stress interfaces support synergy rather than additive effects. Recent research (2023–2025) has shown that nanovesicle-assisted administration significantly enhances the anticancer efficacy of flavonoids by increasing intracellular uptake and stabilizing redox-active molecules. Lipid-based vesicles containing Quercetin or Fisetin significantly reduced breast cancer growth by modulating ROS, SOD/CAT pathways, and NF-κB signaling [1,52]. In the medicinal plant realm, *Ginkgo biloba*, *Hippophae rhamnoides* L., and *Echinacea purpurea* are notable sources of antioxidant properties and immune-boosting effects [1,53]. These data confirm our rationale that increasing the redox stability and intracellular transport of genistein can augment its antiproliferative effects through oxidative stress-proliferation interaction.

Microneedle (MN) arrays have the potential to be employed not only for drug administration, but also for immunization, diagnostic sampling, and targeted therapy in breast cancer treatment [54,55]. MNs consist of micrometer-scale needle structures arranged on a substrate. Needle lengths vary from 150 to 1500 μm, widths from 50 to 250 μm, and tip thickness from 1 to 25 μm [56].Dissolving microneedles (DMNs) are typically produced by casting a drug-loaded polymer solution into a mold, permitting it to dry, and subsequently demolding the solidified microneedle array [57].Common matrix polymers employed in DMN include polyvinyl pyrrolidone (PVP), polyvinyl alcohol (PVA), hyaluronic acid (HA), Chitosan (Ch), sodium alginate (SA), pullulan (PU), eudragit, carboxymethyl cellulose (CMC), chondroitin sulfate (CS), starch, and silk fibroin. These polymers are classified as ductile or brittle materials based on their mechanical properties [58,59]. Dissolving microneedles enhances local efficacy and reduces systemic toxicity, indicating their potential as a valuable adjunct to traditional medicines [59]. They are particularly appropriate for cases involving superficial lesions, skin metastases, or long-term maintenance therapy.

Pullulan (PU) is a hydrophilic exopolysaccharide approved by the FDA [60]. It has neither ionic, hygroscopic, immunogenic, poisonous, carcinogenic, nor mutagenic properties [61]. Pullulan is also an ideal substance for maintaining and regulating various cell types, supporting healthy skin [62]. Additionally, it can promote healing in the area around the injury, help the body’s natural healing process, and maintain hydration [63,64]. We hypothesized that the synergistic anticancer effect of the partial receptor antagonist activity of the natural phytoestrogen GNS and the antimetabolic activity of P.O. would be observed in breast cancer. We investigated such a hypothesis in the current study. The principal goal of the hypothesized combination is to enhance therapeutic efficacy, “competing with endogenous estrogen and dampen estrogen-driven proliferation through multiple pathways in the tumor microenvironment” while reducing systemic toxicity. Two innovative delivery strategies were developed to enhance bioavailability, improve localization of the two active constituents, GNS and P.O., prolong their release, and enhance penetration of these bioactive compounds: a microneedle-based transdermal system and a nanovesicle delivery platform, which increase cellular uptake and stability of the active compounds. The value of our study is that it will give a new hope for the treatment of breast cancer on both the therapeutic and industrial levels.

## 2. Materials and Methods

### 2.1. Chemicals and Reagents

Genistein (GNS) (C_12_H_10_O5, MW: 270.24, CAS: 446-72-0, HPLC purity ≥98%) was obtained from DESITE Biological Technology Co., Ltd. (Chengdu, China). Pullulan (C_23_H_42_O_16_) (MW 574.570 g/mol, purity ≥95%) was purchased from Sigma-Aldrich Co. CAS No.: 9057-02-7 (Saint Louis, MO, USA). Pumpkin seed oil (P.O.) was procured from Haraz Company, Cairo, Egypt, and Brij30 from Alpha Chemika, Andheri, India (CAS number: 8016-49-7). Spectra Por© dialysis membrane tubing (molecular weight cutoff: 14 g/mol) was acquired from Spectrum Laboratories, Inc. (Rancho Dominguez, CA, USA). All other chemicals (HPLC grade) were obtained from Thermo Fisher Scientific (Waltham, MA, USA).

### 2.2. Genistein High-Performance Liquid Chromatography Analysis

Genistein was quantified using High-Performance Liquid Chromatography (HPLC) with a UV-Vis detector (Agilent 1260 Infinity II/DAD; Agilent Technologies, Santa Clara, CA, USA). Separation was done at room temperature with a C18 reverse-phase column (250 mm × 4.6 mm, 5 µm particle size). The mobile phase consisted of methanol, water, and acetic acid in a 60:39:1 volume ratio, with a flow rate of 1.0 mL/min under isocratic conditions. The detection was performed at 260 nm, corresponding to the maximum absorbance of GNS [65,66]. The methodology showed a linear relationship (R^2^ = 0.999). To maintain sink conditions and prevent external auto-concentration, 300 μL aliquots were extracted at regular intervals and immediately replaced with pre-warmed phosphate-buffered saline (PBS, pH 7.4). Total GNS emissions were quantified over time using a standard calibration curve based on known concentrations.

### 2.3. Animals

Female Swiss albino mice (weighing around 23–25 g; 4–6 weeks) were kept in plastic polyethylene cages, five mice per cage (floor area ~1500–1800 cm^2^) and fed a regular diet with unlimited access to food and water at a temperature of 25 °C and a standard light/dark cycle. Before the trial began, the animals were given a one-week acclimatization period. All investigations were conducted in accordance with protocols, and the committee’s policies are based on the National Institute of Health Guidelines for the Care and Use of Laboratory Animals and the ARRIVE standards (Bethesda, MD, USA). We followed the rules outlined in the Guide for the Care and Use of Laboratory Animals, published by the US National Institutes of Health (NIH Publication No. 85-23, revised 2011). Ethical approval was obtained from the Research Ethics Committee (REC) of Cairo University’s Faculty of Pharmacy, which approved this study under the number PI 3850, date of approval 31 March 2025.

### 2.4. Methods

#### 2.4.1. Design of Experiments Genistein-Pumpkisomes (GNS-PKs)

The main aim of the current design was to develop a GNS-PKs formulation under specific conditions, focusing on achieving maximum entrapment efficiency (E.E%) and zeta potential (Z.P), while reducing particle size (PS) and polydispersity index (PDI). During initial testing, independent variables affecting these outcomes were identified. A 3^3^ full factorial design in Design-Expert version 13 (Stat-Ease, Inc., Minneapolis, MN, USA) was employed to examine these variables and their interactions. The selected parameters include P.O. quantity (X1), cholesterol concentration (X2), and Brij 30 concentration (X3), all measured in mg. Table 1 lists the dependent responses: particle size (Y1), polydispersity index (Y2), zeta potential (Y3), and entrapment efficiency percentage (E.E%; Y4).

#### 2.4.2. Preparation of GNS-Loaded Pumpkisomes

**Figure 1:**
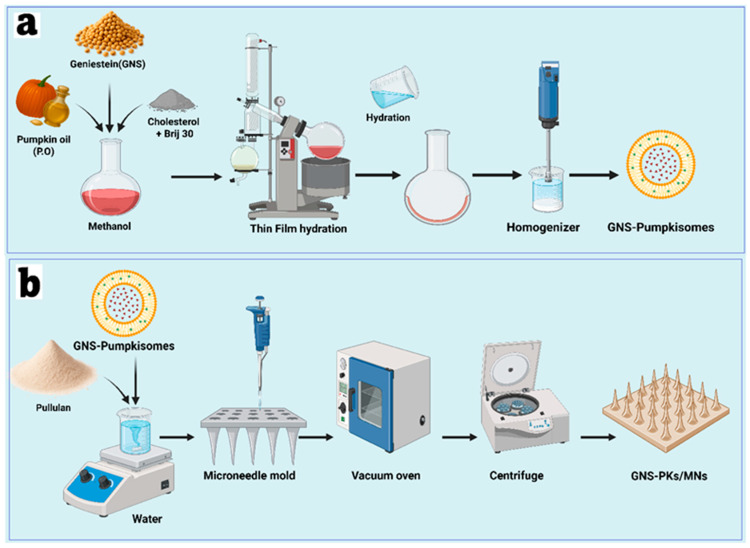
(**a**) Diagram illustrates the methodology for preparing Genistein-Pumpkisomes (GNS-PKs), and (**b**) Diagram illustrates the methodology for fabrication of multifunctional Optimized GNS-PKs-loaded microneedle patches (GNS-PKs/MNs).

Pumpkisomes were produced using the thin-film hydration process. Different amounts of P.O., cholesterol, 20 mg of GNS, and Brij 30 (Table 1) were dissolved in methanol in a round-bottomed flask and then evaporated under vacuum with a rotary evaporator (Perfit Equipment, Ambala, India) to remove residual organic solvent. The dry film in the Rotavaporator was left overnight to eliminate methanol residues and prevent emulsion formation from leftover organic solvent. The dry film was hydrated at room temperature for 30 min using phosphate buffer (pH 7.4), as shown in Figure 1a. The vesicular dispersion was sonicated for 10 min to obtain a uniform size distribution. The dispersion was sealed and cooled to 4 °C overnight before further analysis. The physical appearance, particle size (PS), polydispersity index (PDI), zeta potential, and entrapment efficiency (E.E%) of the formulation were reported.

#### 2.4.3. Evaluation of GSN-Loaded Pumpkisomes

##### Measurement of Particle Size (PS), Polydispersity Index (PDI), and Zeta Potential (Z.P)

The average particle sizes (PS), polydispersity indices (PDI), and zeta potentials (Z.P) of the GNS-PKs-formulated vesicular systems were measured using Photon Correlation Spectroscopy (PCS). Each vesicular dispersion was diluted with about 0.2 µm of filtered distilled water before measurement with a dynamic light scattering device (Brookhaven Instruments Corp., Holtsville, NY, USA). All measurements were conducted in triplicate [67,68].

##### Assessment of Entrapment Efficiency (E.E)

The entrapment efficiency of GNS-PKs was expressed as a percentage. Entrapment efficiency (E.E) was indirectly determined by measuring the amount of unentrapped drugs in the dispersion medium. 3 mL samples from each vesicular dispersion were centrifuged at 20,000 rpm for about 90 min at 4 °C using a high-speed centrifuge (SIGMA 3–30 K, Osterode am Harz, Lower Saxony, Germany). The supernatant was decanted and then diluted with ethanol before analysis of the free drug. The diluted supernatant was analyzed with High-Performance Liquid Chromatography (HPLC) equipped with a UV-Vis detector (Agilent 1260 Infinity II/DAD, Agilent Technologies, Santa Clara, CA, USA) to measure the concentration of unentrapped GNS at 260 nm. To avoid overestimation associated with burst release, E.E% analysis was performed exclusively on the synthesis-derived supernatant before any release experiments. Only unencapsulated GNS was quantified, ensuring that transient desorption or initial burst release did not influence E.E% calculations. The EE% was calculated using Equation (1) provided below [69]:

(1) $$E.E\%=\frac{Total\;GNS\;concentration-Concentration\;of\;unentrapped\;GNS}{Total\;GNS\;concentration}\times 100$$

#### 2.4.4. Optimizing GNS-PKs

The levels of the independent variables were optimized using Design-Expert^®^ software version 7 to minimize P.S and PDI and maximize E.E% and Z.P (absolute values).

#### 2.4.5. Characterization of Optimized Formulae

##### Transmission Electron Microscopy

The morphology of vesicles was assessed using transmission electron microscopy (TEM) (JEM-1400 Plus, Jeol, Tokyo, Japan). Sample preparation entailed applying a single drop of the vesicular dispersion onto a carbon-copper plate. The specimen was allowed to dry before staining with uranyl acetate. The plates were subsequently affixed in the equipment holder for analysis and photomicrography [70,71].

##### Fourier Transform Infrared Spectroscopy (FTIR)

FTIR spectroscopy was used to investigate potential chemical interactions and compatibility among pure GNS, blank GNS, Blank MNs, lyophilized optimum GNS-PKs, and GNS-PKs loaded with Pullulan MNs using an FTIR instrument (Bruker, Coventry, UK). One milligram of each sample was combined with 100 mg KBr and compacted into a tablet. The FTIR spectra were recorded within the region of 4000–400 cm^−1^ at ambient temperature, with a resolution of 4 cm^−1^ and an average of 16 scans [72,73].

##### Differential Scanning Calorimetry (DSC)

To illustrate the thermal properties of pure GNS, blank GNS, Blank MNs, lyophilized optimum GNS-PKs, and GNS-PKs loaded with Pullulan MNs. The DSC thermograms were scanned using a thermal analyzer (TA-60; Shimadzu, Kyoto, Japan). The powders were tightly packed into aluminum pans and heated from room temperature to 400 °C at 10 °C/min [74,75].

##### Effect of Storage on the Optimal GNS-PKs Formulae

The optimized GNS-PKs formula was maintained at 4 °C and ambient temperature for 3 and 6 months, respectively. Physical stability was assessed by comparing E.E% (encapsulation efficiency), particle size (P.S), polydispersity index (PDI), zeta potential (Z.P), and E.E% values before and after storage [76,77]. The statistical analysis was conducted in SPSS (version 26.0; IBM Corp., Armonk, NY, USA) and employed a one-way ANOVA. A *p*-value exceeding 0.05 indicates the absence of a significant difference. The experiment was performed three times, resulting in a standard deviation (SD) of *n* = 3.

#### 2.4.6. Design and Fabrication of Multifunctional Pullulan Microneedle Patches

The microneedle array was created using micromolding with 1 cm × 1 cm polydimethylsiloxane (PDMS) molds (Silicone Template ST-01; Micropoint Technologies Pte Ltd., Singapore). These molds feature a 10 × 10 array of conical holes, 300 μm high, 100 μm wide at the base, and 5 μm at the tip. Optimized microneedles were produced with three pullulan concentrations: M1 (20% *w*/*w*), M2 (30% *w*/*w*), and M3 (40% *w*/*w*), all prepared in ultrapure water. Their viscosity was measured using a Brookfield DV2T rotational viscometer (AMETEK Brookfield, Middleboro, MA, USA). The preparation of the microneedle patch involved two steps: first, 60 μL of pullulan solution was dispensed onto the PDMS mold surface and spread evenly with a spatula; second, the molds were placed in a vacuum oven (Shanghai Jing Hong DZF-6020, Shanghai, China) at 800 mmHg for 10 min to facilitate solution flow into the mold cavities. The molds were then centrifuged for 50 min at 3000 rpm (Beckman, Fullerton, NU, Canada) to ensure thorough degassing of the tips and tamping. Before vacuum penetration and applying the base layer, any excess polymer was removed. To incorporate the GNS-PKs nanoparticles into the pullulan microneedle matrix, the optimized dispersion was carefully mixed with the pullulan solution under low-shear conditions to prevent vesicle disruption. The mixture was stirred at room temperature for 15 min to ensure uniform dispersion, followed by vacuum-assisted degassing to remove trapped air bubbles. This homogeneous solution was cast into silicone microneedle molds and dried under controlled temperature and humidity conditions, ensuring nanoparticles remained suspended within the aqueous polymer network before solidification. Drying conditions for the microneedles were set at 40 °C with 25–30% relative humidity, for 10 h for the tips and 12 h for the base layer, as shown in Figure 1b. A 600 μL pullulan hydrogel was applied to the solidified microneedle tips, followed by centrifugation for 5 min and overnight drying at 40 °C to produce a smooth, bubble-free base layer. Finally, the microneedle patch was released from the mold. Microneedle patches (MNs) containing optimized GNS-PKs and PKs were then fabricated.

#### 2.4.7. Characterization of Multifunctional Pullulan Microneedle Patches

##### Drug Contents

Magnetic agitators containing GNS-PKs/MNs were dissolved in distilled water containing 2.5% Tween 80 and stirred at 300 revolutions per minute for 1 h. The obtained solution was diluted with methanol and sonicated for 5 min to ensure complete dissolution of GNS within the constructed GNS-PKs/MNs. The validated HPLC method described in the preceding section was utilized to conduct this procedure. The drug content of the formed MNs was subsequently confirmed [56,78].

##### Mechanical Strength and Penetration Capability Test

Weights of 250 g, 500 g, and 1000 g were applied to the tips of the GNS-PKs/MNs patch for five minutes, after which they were removed to evaluate the height reduction of the DMN. The DMN was assessed using optical microscopy and fracture analysis, a technique for determining mechanical strength. Parafilm M^®^ (Bemis Company Inc., Neenah, WI, USA), a flexible olefin-based thermoplastic film commonly used to mimic the mechanical properties of human skin, was employed according to Elhabal’s notably modified methodology [54]. An eight-layer film was folded to simulate manual microneedle (MNs) insertion into the skin. MN arrays were pressed against the film at 32 N and 0.1 mm/s [17,18]. The array was then carefully extracted from the Parafilm. After unfolding the layers, the holes made by insertion were measured using a C-B10 + stereomicroscope (Optika, Ponteranica, Italy) and Equation (2). These characteristics enhance the structural integrity of microneedles under applied force and improve their ability to penetrate skin-like substrates.

(2) $$\%\;\mathrm{Penetration}\;\mathrm{capability}=\frac{Number\;of\;holes}{Total\;needles}\times 100\%$$

#### 2.4.8. Characterization of Optimized Microneedle

##### Scanning Electron Microscopy (SEM) Analysis

The morphology of the GNS-PKs/Pullulan MNs patch was investigated using scanning electron microscopy (SEM) (Model SU8010, Hitachi High-Technologies Corporation, Tokyo, Japan), which was used to examine the surface morphology. The microneedle arrays were coated with gold by sputtering, mounted on adhesive metal stubs coated with a 40–60 nm gold layer, and analyzed using a scanning electron microscope (SEM) (Carl ZEISS Pvt. Ltd., Model: EVO LS 15, Cambourne, UK) [79,80]. Digital images were acquired at magnifications of 50×, 150×, 300×, and 800×.

##### Fourier Transform Infrared (FTIR) Spectroscopy

FTIR spectra were obtained for Pullulan/MNs blank without GNS-PKs, and the optimum MNs1 formula, by the same method mentioned before.

##### Differential Scanning Calorimetry (DSC)

DSC was obtained for the Pullulan/MNs blank without GNS-PKs and for the optimum MNs1 formula using the same method described previously.

##### Drug Release Studies

- In Vitro Drug Release Study

The dialysis bag method was employed to examine the release profiles of pure GNS, GNS within optimized GNS-PKs, and optimal GNS-PKs/Pullulan MNs. The dialysis bag was made of cellulose acetate (visking^®^, 28 mm, MWCO 12,000–14,000; Serva, Heidelberg, Germany) [81,82]. The bag was sealed at both ends using suitable clamps after adding 2 mL of PBS containing 5% Tween 20. Bags were placed in 100 mL of 5% *v/v* ethanol in PBS (pH 7.4) to ensure sink conditions and incubated in a thermostatically controlled water bath at 37 ± 0.5 °C with shaking at 100 rpm for 24 h. To prevent evaporation of the release medium, the receptor compartments were sealed. To maintain sink conditions, 2 mL of the sample was collected at various time points (0.5–48 h) and replaced with an equivalent volume of fresh release medium [81,83]. HPLC analysis of the samples was performed at 260 nm. The in vitro drug release study was performed at least 3 times, and the results were analyzed using standard deviation (mean ± SD). The steady-state flux (Jss) and the percentage of drug release after 24 h (Q24%) were determined. The in vitro release data were evaluated kinetically using four distinct release models: zero-order, first-order, and Higuchi. Release studies were conducted in triplicate (*n* = 3).

- Ex Vivo Drug Permeation

Ex vivo permeation studies of GNS from optimal GNS-PKs and optimal GNS-PKs/Pullulan MNs were conducted using the Franz diffusion cell, featuring a diffusional area of 3.14 cm^2^. Ventral rat skin was utilized, with full-thickness skin positioned in the receptor compartment, ensuring the stratum corneum faced the donor chamber. A cylindrical stainless-steel weight (4 g) was applied to the MN arrays for approximately 5 s before removal, facilitating the penetration of the drug-loaded MNs into the skin and placement in the diffusion chamber. The 20 mL PBS receiver compartment (pH 7.4) was degassed and maintained at 37 ± 1 °C. The donor compartment of the diffusion cell was subsequently secured to the receiver compartment. Aliquots of 500 µL were extracted from the Franz cell at consistent intervals and subsequently replenished with pre-warmed PBS. Samples underwent filtration through 0.45 μm filter paper discs, followed by quantification of sitagliptin concentration [55,84,85]. The ex vivo permeation of GNS from both MNs at 24 h was assessed using one-way ANOVA and Tukey’s Multiple Comparison post hoc test, with a significance threshold of *p* ≤ 0.05.

### 2.5. In Vitro Cell Culture Studies

#### 2.5.1. Cell Lines and Culture Conditions

The MCF-7 (ATCC^®^ HTB-22™) human breast adenocarcinoma cell line was obtained from the American Type Culture Collection (ATCC) located in Manassas, VA, USA, through VACSERA Egypt. The cells were maintained in Dulbecco’s Modified Eagle Medium with high glucose, supplemented with L-glutamine, sodium pyruvate, sodium bicarbonate (Sigma-Aldrich), 10% fetal bovine serum (FBS) (Gibco, Grand Island, NY, USA), and antibiotics at 100 units/mL and 100 µg/mL [86,87]. Penicillin and Streptomycin were used under humidified air containing 5% CO_2_ at 37 °C (Thermo Fisher Scientific, USA). Doxorubicin (DOX), a chemotherapeutic fluorescent drug, was obtained from Cadilla Pharmaceuticals Ltd. (Ahmedabad, India). The experimental solution was prepared by dissolving DOX powder in DMSO. The working concentration of the solution employed was 10 M. The cells were resuspended in 1× PBS and combined with Doxorubicin for subsequent analysis [1].

#### 2.5.2. Cellular Cytotoxicity

The cellular cytotoxicity of GNS-free PKs and optimal GNS-PKs was assessed using the MTT assay. Doxorubicin served as a positive control. MCF-7 cells were plated in 96-well plates at a density of 5 × 10^3^ cells per well and incubated at 37 °C with 5% CO_2_ for 24 and 48 h. The medium was then replaced with MTT solution (0.5 mg/mL) and incubated for 3 h. The formazan blue crystals were dissolved in DMSO, and absorbance was measured at 570 nm using a Tecan Infinite F50 ELISA plate reader (Tecan, Männedorf, Switzerland). Optical density was compared with the control, which was set at 100% cell viability. Cell viability percentages and IC50 values were calculated [18].

### 2.6. In Vivo Study

#### 2.6.1. Induction of Breast Cancer in Mice

Female Swiss albino mice were utilized to develop the Solid Ehrlich Carcinoma (SEC) model. Ehrlich Ascites Carcinoma (EAC) cells (ATCC CCL-77), derived from spontaneous murine mammary adenocarcinoma, were expanded intraperitoneally by injecting 100 µL of EAC suspension (2.5 × 10^6^ cells/mouse). Ascitic fluid was collected under sterile conditions, diluted in saline, and viable cells (with >99% viability confirmed by trypan blue exclusion) were enumerated using a Neubauer hemocytometer [1,88]. SEC tumors were induced through the subcutaneous injection of 0.2 mL of viable EAC cell suspension into the left mammary fat pad. Tumor progression was assessed every 48 h with a digital caliper until tumors reached approximately 100 mm^3^, at which time the animals were included in the treatment study. The calculation of tumor volume (V) was performed using the standard formula: V = (length × width^2^)/2. All surgical and handling procedures were performed under anesthesia with ketamine (10 mg/mL) and xylazine (1.25 mg/mL). At the conclusion of the study, tumors were excised, weighed, and subjected to biochemical and histopathological analysis. Tissue homogenates (10% *w*/*v*) were prepared in cold PBS (pH 7.4), centrifuged at 10,000× *g* for 15 min at 4 °C, and the supernatants were stored at −80 °C for further analyses [89,90].

#### 2.6.2. Experimental Design

**Figure 2:**
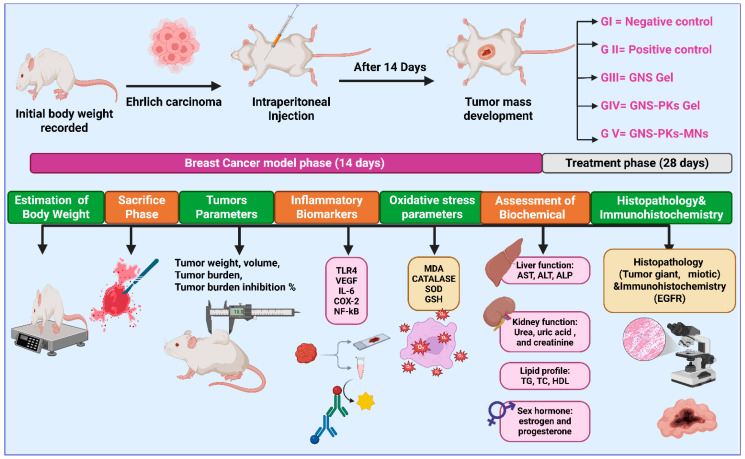
Schematic Representation of the In Vivo Ehrlich Breast Cancer Model and Evaluation Parameters.

Fifty mice were randomly assigned to five groups, with ten mice in each. Group I consisted of healthy negative controls, while Group II included untreated SEC-bearing mice. Group III received SEC treatment with GNS gel (2% *w*/*w* HPMC); Group IV was treated with SEC using the optimal GNS-PKs gel (2% *w*/*w* HPMC); and Group V received optimal GNS-PKs incorporated into pullulan microneedle (MN) patches. HPMC (2% *w*/*w*) was selected as a supporting gel due to its biological inertness and non-irritant profile in skin applications, as shown by multiple studies indicating no inflammatory responses or tissue reactivity [91]. A gel-only control (HPMC without GNS or nanoparticles) showed no therapeutic effect, demonstrating that HPMC did not inhibit tumor growth. A limitation of this study is that preliminary in vitro diffusion experiments revealed delayed release of GNS and GNS-PKs from the gel matrix. In the MN administration process, a single MN array was applied to the left dorsal thoracic skin of each mouse, positioned directly above the tumor-bearing mammary fat pad. MN patches were applied every 48 h for 28 days, totaling 14 applications per animal. Each application delivered a genistein-equivalent dose of 50 mg/kg, resulting in a total dose of 700 mg/kg over the treatment period. The co-delivered unsaturated fatty acids from pumpkin oil were administered at a fixed ratio according to the optimized GNS-PKs formulation. Tumor volumes were measured on Days 0, 7, 14, 21, and 28 to evaluate treatment response over time, rather than relying solely on endpoint measurements. Body weight and health scores were monitored throughout the study to assess systemic tolerability. In vitro release studies indicated sustained GNS release for 48 h. The prolonged in vivo efficacy over 28 days is attributed to (i) repeated MN administration every 48 h, maintaining a steady state of local exposure, and (ii) the observed depot-like retention of lipid nanoparticles in dermal tissue after MN insertion, enabling prolonged residence and gradual diffusion toward the tumor. Prior research has shown extended intradermal retention and slow clearance of lipid-based nanoparticles administered via microneedles, supporting the sustained therapeutic effect observed. Treatments began when tumors reached approximately 100 mm^3^, continued for 28 days, and were administered transdermally every other day, as shown in Figure 2. MN patches were applied to the left dorsal thoracic skin, immediately above the tumor-bearing mammary fat pad. All formulations delivered an equivalent of 50 mg/kg GNS [14]. The 50 mg/kg genistein-equivalent dose was chosen based on previous pharmacokinetic and efficacy studies that demonstrated adequate exposure at 25–100 mg/kg without significant effects [92,93].

#### 2.6.3. Estimation of Skin Morphology and Body Weight

To evaluate morphological changes in the skin above and around subcutaneous tumors, such as epidermal/dermal architecture, ulceration, inflammation, vascular/lymphatic remodeling, fibrosis, and tumor-skin interface features. Body weights were measured for all animal groups right before the study and at the conclusion of the 4-week study using an electronic balance (Sartorius BL210S, Sartorius AG, Göttingen, Germany) [91,92], as illustrated in Figure 2.

#### 2.6.4. Estimation of Tumor Parameters

A Digital Vernier Caliper was used to measure the tumor size, and was quantified using the following formula [1,93]:

To calculate tumor volume (cm^3^), use Equation (3):

Tumor volume = length × (width)^2^ × 0.5 (3)

The tumor load was determined in the following Equation (4):

Tumor burden = ∑ tumor mass in a group(4)

Tumor burden reduction was determined in the following Equation (5):

(5) $$\%\;\mathrm{Tumor}\;\mathrm{burden}\;\mathrm{inhibition}=\frac{Tumor\;burden\;in\;Ehrlich\;tumor-burden\;in\;the\;test\;group}{Tumor\;burden\;in\;Ehrlich\;tumor}\times 100$$

Tumor weight measured by an electric balance expressed in grams unit.

#### 2.6.5. Assessment of Inflammatory Biomarkers

The anti-inflammatory and anti-proliferative effects of genistein treatment in breast cancer were assessed by measuring key tissue biomarkers using ELISA kits (MyBioSource, DuoSet^®^, San Diego, CA, USA) according to the manufacturer’s guidelines. The selected biomarkers were chosen for their mechanistic relevance to the known biological effects of GNS, particularly its impact on inflammatory, proliferative, and angiogenic pathways. Analyze tissue homogenates for the levels of TLR4 (Toll-Like Receptor 4), VEGF (vascular endothelial growth factor), IL-6 (interleukin-6), COX-2 (cyclooxygenase-2), and NF-κB (nuclear factor kappa B) [94,95,96], as illustrated in Figure 2.

#### 2.6.6. Assessment of Oxidative Stress Parameters

Catalase (CAT) activity was assessed by measuring the rate of hydrogen peroxide (H_2_O_2_) degradation at 240 nm, according to established protocols [88,89]. Mammary tissues were homogenized in phosphate-buffered saline (PBS; pH 7.4; 8% *w*/*v*) and subsequently centrifuged at 15,000× *g* for 20 min at 4 °C. Results were expressed as units per milligram of protein. Absorbance measurements were obtained at 520 nm [1,97].

Superoxide dismutase (SOD) activity was assessed using the method developed by Nandi and Shubham, which measures the enzyme’s ability to inhibit the auto-oxidation of pyrogallol in alkaline conditions [98,99]. The reaction mixture comprised 2 mL of 50 mM Tris-cacodylate buffer at pH 8.5. The reaction was initiated by adding 100 µL of freshly prepared 6 mM pyrogallol in 10 mM HCl to a buffer containing 20 µL of tissue homogenate. Absorbance was recorded at 440 nm over a duration of 2 min. SOD activity was quantified as units per milligram of protein, with 1 unit defined as the amount of enzyme required to inhibit 50% of pyrogallol auto-oxidation [100].

Reduced glutathione (GSH)levels were assessed using a traditional DTNB-based colorimetric method. One milliliter of the tissue sample was combined with 1 mL of 5% trichloroacetic acid (TCA) and centrifuged at 1200× *g* for 20 min. Following this, 0.5 mL of the supernatant reacted with 2 mL of 5,5′-dithiobis (2-nitrobenzoic acid) (DTNB). The resultant yellow product exhibited an absorbance at 412 nm. GSH content was quantified as nmol/mg of protein [101,102].

#### 2.6.7. Assessment of Kidney Function, Liver Function (LFTs) Biomarkers, and Sex Hormones

Renal biomarkers, including urea, uric acid, and creatinine, as well as total bilirubin and the activities of aspartate aminotransferase (AST), alanine aminotransferase (ALT), and alkaline phosphatase (ALP), were quantified using commercial reagent kits and a spectrophotometric analyzer. Serum concentrations of estrogen and progesterone were measured using standard commercial assays following the manufacturer’s instructions [1].

#### 2.6.8. Lipid Profile

Plasma triglycerides (TG) were quantified using the method established by Rice (1970) [103]. Total cholesterol (TC) and High-density lipoprotein (HDL) cholesterol were quantified using the methodology described by Burstein et al. (1970) [104,105].

### 2.7. Histopathologic Analysis

Histopathological examination was performed on 5 μm-thick paraffin sections following staining with hematoxylin and eosin (H&E) [106,107]. The examination used a photomicroscope to obtain images at ×100 magnification (Olympus BX51, Olympus America, Melville, NY, USA). Sections were analyzed for alterations, including the presence of tumor giant cells and mitotic figures. The mean ± SD of the average number of mitotic figures and giant tumors per five high-power fields (HPFs) was calculated and reported [1].

### 2.8. Immunohistochemical (IHC) Examination of Epidermal Growth Factor Receptor (EGFR)

Breast tissue samples were processed for immunohistochemical evaluation according to the manufacturer’s instructions using an IHC staining kit. Antibodies against EGFR (5B7, Rabbit monoclonal, Roche Diagnostics) were applied at a 1:100 dilution, and each group was incubated overnight according to the manufacturer’s guidelines. Marker expression was visualized using peroxidase and diaminobenzidine (DAB; Sigma) to detect the antigen–antibody complex. Negative controls utilized non-immune serum as a substitute for the primary or secondary antibodies [108,109]. IHC-stained sections were examined using an Olympus microscope (BX-63) (Olympus Corporation, Tokyo, Japan). Immunohistochemistry results were scored by quantifying the percentage of reaction area in 7 microscopic fields using ImageJ (version 1.53t; National Institutes of Health, Bethesda, MD, USA) 1.53t, developed by Wayne Rasband and contributors at the National Institutes of Health, USA, at magnifications of 100× (100 µm) and 400× (25 µm).

### 2.9. Statistical Analysis

Group differences were assessed using one-way ANOVA, complemented by Tukey’s post hoc test, using GraphPad Prism 8.0 (*p* < 0.05 considered significant). Tumor volume, tumor weight, and biochemical parameters were analyzed similarly using one-way ANOVA followed by Tukey’s multiple-comparison test. A two-way repeated-measures ANOVA was employed to analyze the changes in body weight and tumor volume over time. Results from both in vitro and ex vivo studies are reported as mean ± SD (*n* = 10). The notation employed is as follows: ns = not significant (*p* > 0.05), * (*p* < 0.05), ** (*p* < 0.01), *** (*p* < 0.001), and **** (*p* < 0.0001).

## 3. Results and Discussion

### 3.1. Development of Genistein-Pumpkisomes (GNS-PKs) Formulations

Pumpkisomes (PKs), as a novel vesicular system prepared from Pumpkin seed oil (P.O.), were inspired by ufasomes, which are unsaturated fatty acid vesicular structures composed of oleic or linoleic acid. Since P.O. is rich in these two unsaturated fatty acids, it was selected for the synthesis of PKs. PKs were formed using a modified thin-film hydration method with P.O., cholesterol, and Brij 30. Cholesterol has previously been reported to increase the E.E% of drugs in ufasomes by altering vesicle fluidity and elasticity. Also, it was proposed to introduce lattice imperfections to create sufficient space to encapsulate drug molecules.

**Figure 3:**
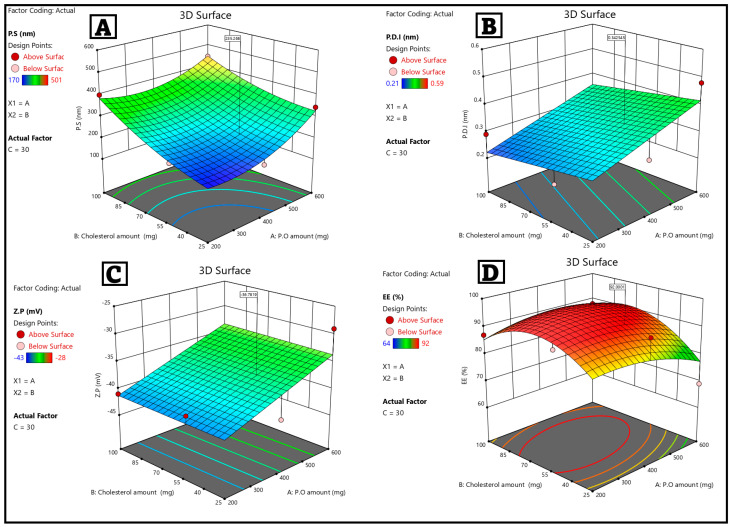
3D Response surface plot showing effect of P.O. amount (**X1**), cholesterol amount (**X2**), and Brij 30 amount (**X3**) concentrations on (**A**) Particle size (P.S), (**B**) polydispersity index (PDI), (**C**) zeta potential (Z.P), and (**D**) Entrapment efficiency percentage (E.E%).

The I-Optimal response surface design elucidated the impact of formulation variables, specifically P.O. amount (A), cholesterol content (B), and Brij 30 concentration (C), on the physicochemical properties of the developed GNS-PKs. Table 2 and Figure 3 show that the measured responses, particle size (PS), polydispersity index (PDI), zeta potential (Z.P), and entrapment efficiency (E.E%), varied considerably between formulations. This shows that the vesicular system is sensitive to compositional changes.

### 3.2. Evaluation of FIS-NSs Formulations (Particle Size, Polydispersity Index, and Zeta Potential)

The particle size varied from 170 ± 0.98 nm (Run 1) to 501 ± 0.46 nm (Run 17), indicating a significant dependence of vesicle formation on the relative proportions of P.O., cholesterol, and Brij 30. The P.O. amount (factor A) demonstrated a direct effect of increasing size at elevated levels. Formulations with 600 mg P.O. (Runs 3, 4, 9, 11, 12, 13, 16, 17) consistently yielded larger vesicles (343–501 nm), presumably attributable to the increased availability of lipid material, which enhances bilayer thickness and vesicle diameter. Reduced P.O. amounts (200–400 mg) led to smaller vesicles, particularly in Runs 1, 6, 10, 14, and 19, suggesting that lower lipid content limits vesicle expansion, and cholesterol influenced size, resulting in alterations. Increased cholesterol levels (100 mg) led to greater vesicle stiffness, resulting in a slight increase in their size (Runs 3, 7, 8, 11, 15, 17). Low cholesterol levels (25 mg) increased bilayer flexibility, resulting in smaller diameters (Runs 1, 5, 13, and 14). Brij 30, a nonionic surfactant, altered the structural composition of vesicles. Brij 30 (30 mg) enhanced the quantity of smaller, more uniform vesicles (Runs 1, 2, 3, 13, and 15), indicating that the surfactant reduces interfacial tension. Runs 6–8, 10–11, 14, 16, and 19 exhibited increased diameters when Brij 30 was administered at a reduced dose of 10 mg. The observed outcome is likely attributable to the instability of the vesicle production process. The shortest PS (170 nm in Run 1) was obtained with a moderate P.O. (400 mg), low cholesterol (25 mg), and high Brij 30 (30 mg), showing that this combination supports optimal vesicle curvature and stability.

PDI values ranged from 0.21 ± 0.03 to 0.59 ± 0.01, indicating that some formulations produced monodisperse systems, while others did not. Lower PDI values (<0.30) correlated with increased surfactant concentrations (30 mg Brij 30), as demonstrated in Runs 1, 2, and 15. This indicates that vesicle assembly has been effectively stabilized. Formulations with higher PDI values (>0.45) were observed in those with increased P.O. or decreased surfactant concentration (Runs 4, 9, 11, 14, 16), indicating structural heterogeneity due to insufficient stabilization or excessive lipid crowding. The findings are consistent with prior studies on fatty-acid vesicular systems, indicating that surfactant concentration plays a crucial role in uniformity.

The zeta potential of all formulations ranged from −28 to −43 mV, indicating significant stability as colloidal systems. Run 14 exhibited the lowest P.O., cholesterol, and Brij 30, resulting in the lowest Z.P of −43 mV. In formulations containing high lipid concentrations (600 mg P.O.) or excessive cholesterol (100 mg), Z.P exhibited a decrease in negative charge, ranging from −28 to −30 mV. The dense bilayer packing likely protected the surface charge. The persistently low Z.P values indicate that GNS-PKs are more effective in conditions of high electrostatic repulsion, which prevents vesicle aggregation similar to the behavior observed in unsaturated lipid vesicles.

The entrapment efficiency (E.E%) ranged from 64% to 92%, indicating effective interaction between the drug and the lipid system. Runs 1 and 14 demonstrated the best energy efficiency rates, over 90%. Each of these trials administered 200–400 mg orally, used a minimum of Brij 30, and demonstrated low to moderate cholesterol levels.

The formulations likely enabled the formation of the flexible bilayer, providing adequate space for the medication within the vesicle matrix. Formulations with supplementary P.O. intake of 600 mg or more cholesterol (100 mg) (e.g., Runs 7, 8, 9, 11, 16, 17) led to a decreased E.E% (64–68%).

At elevated lipid densities, vesicular membranes exhibit reduced permeability and increased rigidity, thereby impeding proper drug absorption. Well-balanced lipid-surfactant compositions (runs 6, 10, 19, and 20) exhibited a moderate encapsulation efficiency (E.E%) ranging from 78% to 89%. The findings are consistent with the known effects of cholesterol on vesicle permeability: increased cholesterol reduces bilayer flexibility, thereby decreasing drug entrapment efficiency.

#### 3.2.1. Statistical Analysis of the Factorial Experimental Design Genistein–Pumpkisomes (GNS-PKs)

The particle size ranged from 170 to 501 nm, showing a notable correlation with the formulation content, as presented in Table 3. The quadratic model yielded an accurate prediction of the response, evidenced by a significant F-value of 16.431 (*p* = 0.0023). An R^2^ score of 0.806 and a precision of 7.421 demonstrate the model’s reliability and an advantageous signal-to-noise ratio. The adjusted R^2^ of 0.632 exceeds the expected R^2^ of 0.458, indicating moderate predictive ability and adequate model stability. Factor B (cholesterol) has been identified as the key determinant of PS. The increase in cholesterol led to a stiffer bilayer, facilitating the formation of larger vesicles, whereas a decrease in cholesterol permitted the formation of more flexible, compact structures. This is consistent with previously reported effects of cholesterol on membrane rigidity and thickness. PDI values ranged from 0.21 to 0.59, indicating varying levels of uniformity across formulations (Table 3). A linear model fit the data well (F = 8.555, *p* = 0.009). The model has a decent fit, as indicated by the R^2^ value of 0.496 and modified R^2^ of 0.401. The estimated R^2^ value of 0.220 suggests limited predictive accuracy, likely due to slight differences caused by the specified factors. Adequate precision (8.428) indicates a strong analytical signal. A and C (P.O. amount and Brij 30) are essential considerations. The P.O. quantity influenced lipid availability, while Brij 30 stabilized the vehicles. Increased surfactant concentrations generally enhanced homogenization, resulting in reduced PDI values, whereas insufficient surfactant or excessive P.O. led to greater heterogeneity [110,111].

Zeta potential varied between −28 and −43 mV, demonstrating excellent colloidal stability in all formulations, as presented in Table 3. A linear model analogous to PDI indicated a response (F = 9.132, *p* = 0.008), with R^2^ = 0.383 and adjusted R^2^ = 0.268, suggesting a modest fit, predicted R^2^ = 0.0476, suggesting restricted accuracy in model predictions. Adequate precision is 5.226, indicating acceptable yet comparatively lower signal strength, with Factor A (P.O. amount) significantly influencing Z.P. Increasing the P.O. amount decreased the net negative charge, likely due to changes in packing density or surface lipid rearrangement. In contrast, lower P.O. Higher levels led to a more pronounced negative zeta potential. The model statistics, while constrained, consistently yield high negative values, indicating robust stability and a low probability of aggregation. The entrapment efficiency ranged from 64% to 92%, indicating a notable complementarity between the medication and the lipid matrix, as illustrated in Table 3. The quadratic model for E.E% yielded a notable F-value of 6.817 (*p* = 0.025), along with an R^2^ of 0.767 and an adjusted R^2^ of 0.558, indicating a substantial degree of model fit. The expected R^2^ value of 0.194 indicates a moderate level of predictive effectiveness. A precision value of 6.311 signifies a reliable signal, with factors A and C identified as significant contributors. The increase in P.O. amounts may have led to greater bilayer rigidity, thereby decreasing encapsulation capacity. Conversely, optimizing Brij 30 concentrations appears to have improved bilayer fluidity and drug accommodation. The presence of excess surfactant or lipid disrupts bilayer packing, leading to a decrease in encapsulation efficiency at extreme factor levels in the design. The quadratic and linear models used for the responses demonstrated significant predictive accuracy in the context of formulation optimization. The identification of cholesterol as the main factor influencing particle size, along with P.O. and Brij 30 as significant modulators of PDI, Z.P, and E.E%, aligns with the known characteristics of lipid–surfactant nanovesicle systems.

#### 3.2.2. Optimization of (GNS-PKs) Formulations

Based on the desirability criterion, Design-Expert^®^ software (Version 13, Stat-Ease Inc., Minneapolis, MN, USA) was used to determine the optimal values of the independent variables. Optimized formulation (Run 1) successfully met all design goals: minimal particle size and P.D. The maximal zeta potential and entrapment efficiency. The design revealed significant interactions between the three formulation variables. P.O. amount predominantly controlled vesicle size and internal volume. Cholesterol modulated membrane rigidity, thereby affecting PS, Z.P, and E.E%, and Brij 30 was critical for stabilizing vesicles and reducing PDI. The overall trends closely resemble those reported for Pumpkisomes and fatty-acid vesicles, reinforcing the suitability of the chosen lipid components for the formulation of GNS-PKs. High entrapment efficiency, nanoscale size, and strong negative zeta potentials across optimized runs demonstrate that the system is promising for further pharmaceutical development. The adequate precision values (>4 for all responses) confirmed sufficient model discrimination, while the F- and *p*-values confirmed the statistical significance of factor effects [46,112]. Although predicted R^2^ values were modest for some responses, the overall model reliability was acceptable for guiding the selection of an optimized R1 formulation with desirable nanoscale size, uniformity, high entrapment efficiency, and stability.

#### 3.2.3. Selection of the Optimum (GNS-PKs) Formulations

R1, composed of 400 mg P.O., 25 mg cholesterol, and 30 mg Brij 30, revealed the highest desirability. Thus, further characterization was adopted for R1.

### 3.3. Characterization of Optimized Formula

#### 3.3.1. Transmission Electron Microscopy (TEM)

**Figure 4:**
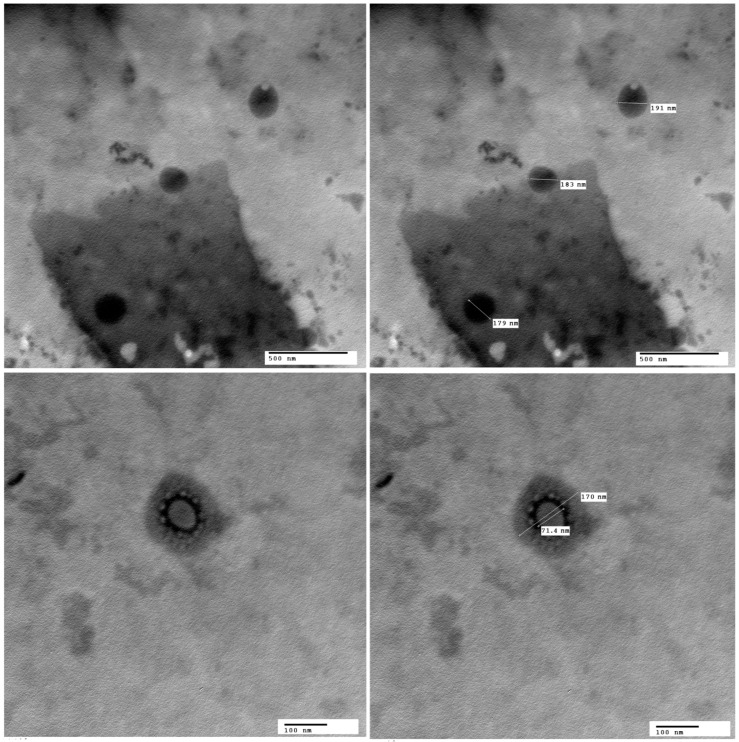
TEM micrograph of optimized Genistein–Pumpkisomes (GNS-PKs) (R1).

Transmission electron micrograph of R1 revealed homogenous non-accumulating spherical nano-sized vesicles (Figure 4), indicating that the majority of the nanovesicles exhibit a spherical morphology with smooth, well-defined edges [113,114]. The vehicles exhibited clear separation, with no signs of aggregation or fusion, indicating successful stabilization of the system, whose average PS was comparable to that recorded with the Malvern Zetasizer Nano Z.S (Table 2).

#### 3.3.2. Fourier Transform Infrared Spectroscopy (FTIR)

**Figure 5:**
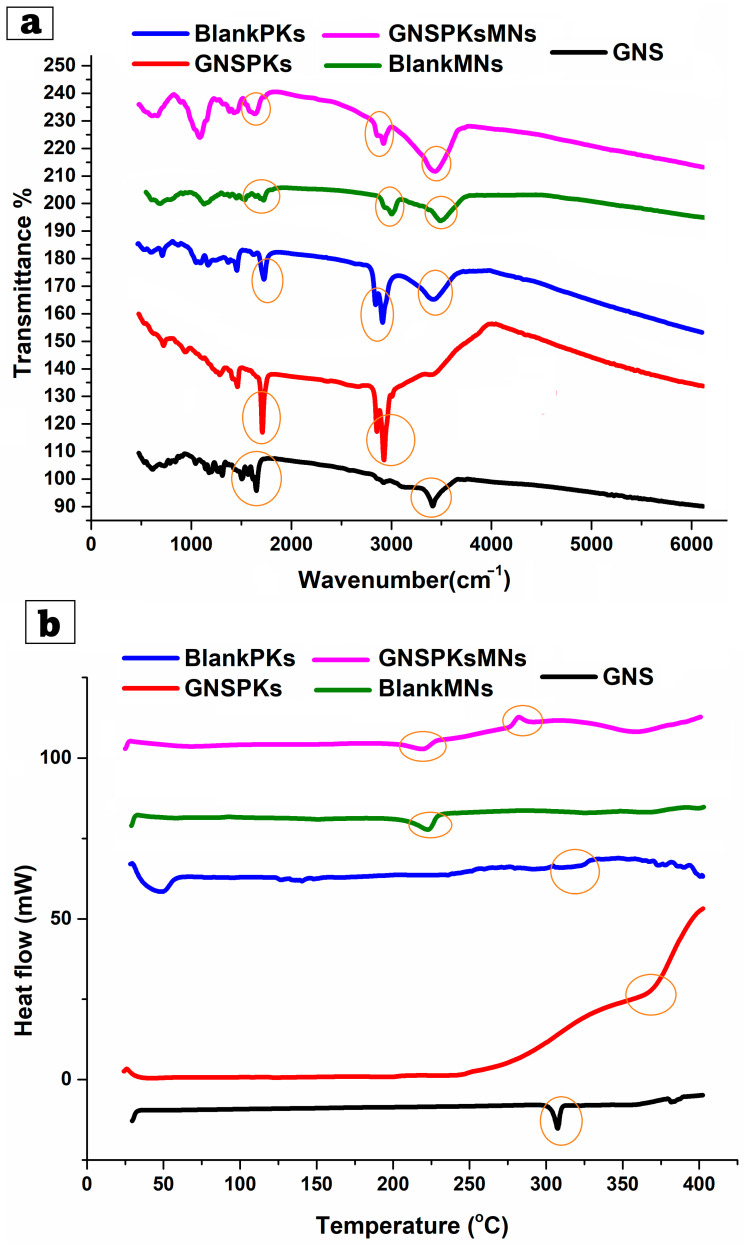
(**a**) Fourier Transform Infrared Spectroscopy, and (**b**) Differential Scanning Calorimetry for pure genistein (Pure-GNS), genistein-loaded pumpkisomes (GNS-PKs), blank pumpkisomes (PKs-Blank), Blank Pullulan MNs (Blank-MNs), and GNS-PKs-loaded Pullulan MNs. Data were expressed as Mean ± SD, *n* = 3. Differences between groups were indicated using orange circles.

FTIR spectroscopy was utilized to evaluate the potential chemical interactions among GNS, the pumpkisomes formulation (GNS-PKs), and the blank pumpkisomes (Blank-PKs), as illustrated in Figure 5a. The FTIR spectrum of pure GNS showed several distinctive peaks confirming its structure. A broad band at ~3400 cm^−1^ indicates O-H or N-H stretching. Firm peaks in the ~2900–2950 cm^−1^ range indicate aliphatic C-H stretching. The absorption near ~1650–1700 cm^−1^ is attributed to C=O stretching (carbonyl group). Bands at ~1500–1600 cm^−1^ indicate aromatic or amide stretching, while peaks at ~1000–1300 cm^−1^ indicate C-O and C-N stretching. Blank-PKs’ FTIR spectrum showed saturated lipid chain-like aliphatic C–H stretching bands at 2850–2920 cm^−1^. A faint band at ~1730 cm^−1^ reflects ester C=O stretching from fatty acids, whereas the broadening in the 1000–1200 cm^−1^ region is due to surfactant C–O–C stretching [115,116]. The absence of GSN peaks suggests that Blank-PKs are entirely lipid–surfactant. GNS-PKs had the lipid peaks of Blank-PKs in their FTIR spectra, but the prominent GNS peaks were reduced or broadened. The C=O stretching band (1650–1700 cm^−1^) exhibited diminished intensity, the N–H/O–H region (~3400 cm^−1^) widened, and the fingerprint region displayed a slight shift in C–O/C–N peaks [117]. The alterations signify GNS encapsulation within the bilayers of pumpkisomes. The attenuation or concealment of GNS’s characteristic peaks indicates that the drug is molecularly dispersed or integrated within the lipid matrix rather than being crystalline or adsorbed. The absence of new peaks signifies a lack of chemical interaction, implying that physical trapping, rather than covalent bonding, governs encapsulation.

#### 3.3.3. Differential Scanning Calorimetry (DSC)

DSC thermograms were used to evaluate the thermal behavior of pure GNS blank pumpkisomes (Blank-Pks), and drug-loaded pumpkisomes (GNS-PKs). The thermal transitions provide insight into drug crystallinity, physical state, and compatibility with excipients. The DSC curves for all samples are shown in Figure 5b. The DSC thermogram of pure GNS showed a sharp endothermic peak at ~300 °C, characteristic of its melting point, confirming the drug’s crystalline nature. This peak serves as a reference for assessing whether GNS remains crystalline or becomes amorphous following encapsulation into pumpkisomes; a sharp endothermic event denotes high purity and crystallinity [118]. Loss or reduction of this peak in formulations indicates successful molecular dispersion. The thermogram of the Blank-PKs showed a broad, smooth thermal profile, with no distinct endothermic transitions. This indicates the non-uniform or partially ordered characteristics of the lipid–surfactant matrix. The lack of pronounced peaks indicates the intrinsic adaptability of the vesicular membrane, a crucial factor for effective drug encapsulation and stability. The thermogram of GNS-PKs did not exhibit any observable melting peak of GNS in the characteristic region around 300 °C [42,119]. A significant thermal shift was noted beginning at approximately 250 °C, with an increase in intensity up to 400 °C. The absence of the GNS crystalline melting peak indicates that the drug has been effectively encapsulated in an amorphous or molecularly dispersed form within the lipid bilayer. Encapsulation interferes with the drug’s initial crystal lattice structure, thereby inhibiting recrystallization. This further proves that drug crystallites are not present on the vesicle surface. This behavior clearly suggests a thorough integration of GNS into the pumpkisomes bilayer.

#### 3.3.4. Stability Study of the Optimized Genistein-Punpkisomes (GNS-PKs)

Table 4 showed that the stability of the improved GNS-PKs formulation was assessed for 6 months at both refrigerated (4 ± 1 °C) and ambient (25 ± 2 °C) temperatures. The initial formulation, with 400 mg pumpkin oil, 25 mg cholesterol, and 30 mg Brij 30, showed a mean particle size of 170 ± 0.98 nm, PDI of 0.27 ± 0.05, Z.P of −42 ± 0.87 mV, and E.E of 92 ± 0.67%. This indicates the formation of a homogeneous, stable nanosystem with a high drug-loading capacity. After 24 h of storage under both conditions, all parameters showed negligible changes, indicating the short-term physical stability of GNS-PKs. The particle size increased progressively at both temperatures after 3 and 6 months of storage. After six months, the particle size increased considerably to 193 ± 0.93 nm under refrigeration and 198 ± 0.98 nm at ambient temperature. With increasing particle size, PDI values increased modestly, particularly under ambient conditions, reaching 0.48 ± 0.05 at 6 months, indicating minimal vesicle aggregation or fusion. The PDI remained within the acceptable range, indicating that the vesicle population was largely homogeneous. Over six months, zeta potential measurements showed a slight decrease in magnitude, with Z.P dropping from −42 mV to −37 mV (refrigerated) and −35 mV (ambient). Electrostatic stability and a high negative surface charge are required to limit vesicle agglomeration during storage. The modest Z.P reduction that did not cause instability can be attributed to either the storage medium or progressive surface modifications. Throughout the trial, entrapment efficiency remained good but decreased with storage time. After six months, E.E values in both storage settings approached 80%, indicating good drug retention with negligible leakage. Prolonged storage, particularly with lipid-based nanocarriers, can cause structural rearrangements or lipid phase transitions, resulting in a modest drop in encapsulation efficiency [120,121]. Refrigeration exhibited superior stability relative to ambient conditions across all metrics, as evidenced by smaller increases in particle size and PDI, along with greater retention of zeta potential and entrapment efficiency. This supports the notion that lower temperatures reduce kinetic energy and chemical activity, thereby inhibiting vesicle fusion and drug efflux. The optimized GNS-PKs formulation demonstrated substantial stability over 6 months, indicating its potential for preclinical or clinical applications. Statistical analysis using one way ANOVA at 95% confidence interval (*p*-value = 0.05) was performed on the results obtained from stability study performed on the improved GNS-PKs formulation at 24-h point, 3 months point and 6 months point at both refrigerated and ambient temperature. Results of particle size, PDI, Zeta potential and encapsulation efficiency showed that there is no significant difference between the three studied points at refrigerated conditions indicating that refrigerated storage maintains physical and chemical qualities.

### 3.4. Characterization of Multifunctional Pullulan Microneedle Patches

#### 3.4.1. Drug Content

Table 5 shows the physical characteristics, drug content, and mechanical performance of the formulated pullulan microneedles (M1–M3). All formulations were successfully produced, exhibiting uniform conical shapes and a consistent drug loading of 2 mg genistein per patch. An increase in pullulan concentration from 20% to 40% *w*/*w* led to notable variations in needle morphology, drug content, and mechanical properties. The rise in pullulan concentration was significantly associated with increased drug content. M1 had a drug level of 72.4 ± 1.5%, whereas M3 had a level of 93.1 ± 0.9%. The observed improvement is due to the higher polymer density in M3, which effectively reduces drug diffusion during drying. Improved interaction between pullulan chains and genistein increases entrapment efficiency, reduces polymer shrinkage, and reduces medication loss from needle tips. As a result, M3 demonstrated improved drug encapsulation and consistency, making it more suitable for applications requiring high-dose or controlled-release delivery.

#### 3.4.2. Mechanical Strength and Penetration Capability Test

**Figure 6:**
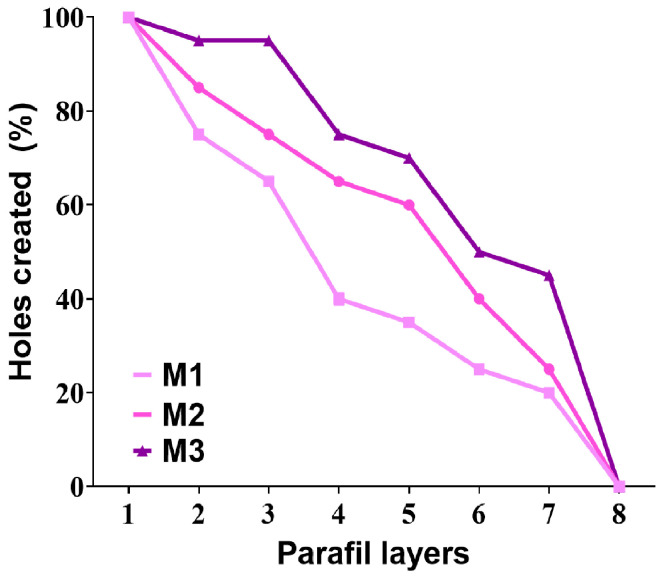
Penetration capability for different microneedles (M1, M2, and M3). **Note:** M1: microneedle containing 20% *w*/*w* pullulan solutions; M2: microneedle containing 30% *w*/*w* pullulan solutions; M3: microneedle containing 40% *w*/*w* pullulan solutions; and all formulation containing 2 mg of genistein. Data were expressed as Mean ± SD, *n* = 3.

Mechanical tests revealed a significant increase in microneedle toughness with increasing pullulan content (Table 5). The measured MNs lengths ranged from 285 to 298 µm, closely matching the initial mold height of 300 µm. This shows that mold filling is effective and structural shrinkage is controlled. M1 with 20% pullulan produced the smallest needles at 285.0 ± 0.6 µm, while M3 with 40% pullulan produced the longest needles at 298.0 ± 0.7 µm. The observed increase in needle length with polymer concentration suggests that concentrated pullulan solutions exhibit higher viscosity and better mold-retention capability. This property is critical to maintaining the structural integrity of the microneedles during drying. The measured tip diameter values (4.0–5.0 µm) confirm that all formulas produced sharp tips suitable for efficient skin penetration. M1 had the smallest tip diameter of 4.0 ± 0.8 µm, whereas M3 had somewhat larger tips measuring 5.0 ± 0.6 µm. This discrepancy is most likely due to increased viscosity, leading to a thicker apex. All tip sizes remained below the commonly accepted threshold of 10 µm for successful insertion. The base diameter increased from 65.0 ± 0.9 µm in M1 to 85.0 ± 1.2 µm in M3. A larger base enhances structural support, indicating that higher pullulan concentrations facilitate the formation of mechanically stable microneedles with reduced susceptibility to bending and buckling. M1 exhibited the greatest reduction in length across all stress levels, indicating its mechanical instability due to low polymer concentration and network density. M2 demonstrated significant resistance to compression and a longer lifespan. The M3 exhibited minimal shape alteration and maintained a majority of its original height under maximum force (1000 g). The findings indicate that the 40% pullulan matrix possesses a robust, cohesive, and mechanically strong structure capable of withstanding manual application stress on the skin [122,123]. The results demonstrate that increased pullulan concentration improves microneedle quality across various parameters. Increasing viscosity enhances mold filling and geometric consistency, whereas increasing polymer density improves the effectiveness of drug encapsulation. Improved structural integrity enhances strength and reduces the likelihood of deformation. M3 (40% pullulan) consistently exhibited enhanced performance across multiple metrics, including needle length maintenance, tip stabilization, base support, drug content, and compression resistance. The identified traits enhance drug insertion and loading, ensuring reliability in clinical applications, thereby positioning M3 as the preferred option for delivering genistein-loaded multifunctional microneedles. A model consisting of eight layers of Parafilm M^®^ was employed to evaluate the insertion performance of pullulan microneedle formulations (M1–M3). Figure 6 presents the proportion of holes formed in each layer. All microneedle patches effectively penetrated the initial Parafilm layer, resulting in 100% penetration. This finding demonstrates that all formulations have adequately sharp tips for effective skin penetration [44]. Changes dependent on the formulation were observed as the layer increased in depth. The optimal penetration level was achieved with M3, which contained 40% pullulan. M3 constituted over 90% of the holes in layer 2, nearly 75% of the holes in layer 4, and more than 40% of the holes in layer 6. The enhancements in M3’s mechanical strength, needle rigidity, and base diameter effectively mitigate insertion bending and blunting. Increased pullulan concentrations result in a thicker polymer network that can withstand insertion forces without distortion. M2, which contains 30% pullulan, showed higher penetration than M1 but was outperformed by M3. M2 exhibited an 80% penetration rate at layer two and 60% at layer four, indicating a satisfactory, albeit suboptimal, level of mechanical resistance. M2 exhibits notable needle sharpness and structural integrity; however, its intermediate polymer composition leads to reduced stiffness compared to M3, rendering it more susceptible to compression during deeper insertions. The M1 formulation, comprising 20% pullulan, exhibited the lowest efficacy, with penetration decreasing from 100% in the first layer to approximately 65% in the third layer and around 40% in the fourth layer. Following layer five, there was a marked reduction in penetration efficiency. The rapid decrease can be ascribed to M1’s diminished mechanical strength, polymer density, and needle deformation under compressive loads. M1 SEM images reveal reduced base thickness and diminished tip support, suggesting a limited number of insertion opportunities. The Parafilm insertion statistics indicate a significant correlation between pullulan concentration and penetration efficacy, with M3 demonstrating superior performance compared to the other alternatives. Pullulan has demonstrated the ability to enhance needle stiffness and structural integrity, thereby facilitating deeper, more uniform penetration into skin-like surfaces [43,56]. The data indicate that formulation M3 is the most effective for transdermal genistein distribution utilizing dissolving microneedle systems.

### 3.5. Characterization of Optimized Microneedle

#### 3.5.1. Morphological Investigation

**Figure 7:**
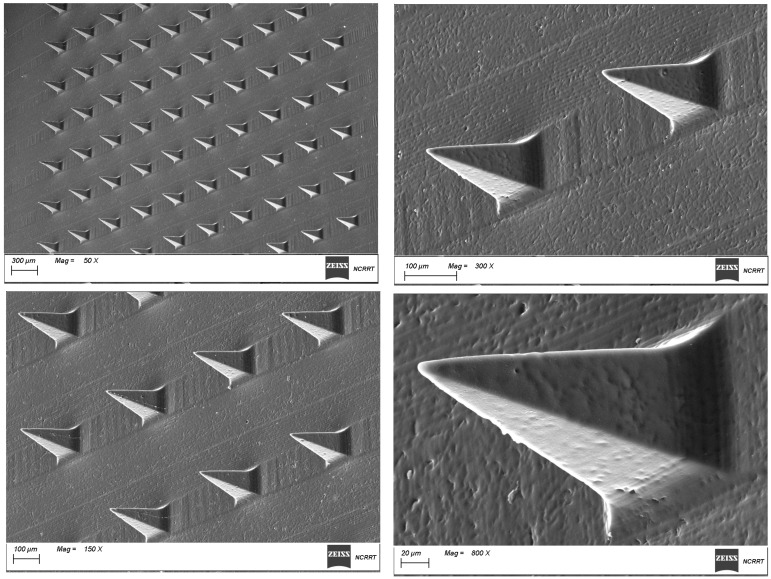
Scanning electron microscopy of optimized microneedles (M3), which are Genistein–Pumpkisomes-loaded Pullulan microneedles (GNS-PKs-MNs).

Figure 7 show morphological investigation (SEM); confirmed that the fabricated pullulan microneedles exhibited optimal morphology: sharp conical tips, smooth surfaces, uniform arrangement, and strong integration with the base layer. These structural characteristics strongly support the microneedles’ ability to achieve reliable skin insertion and consistent drug delivery performance for breast cancer [55].

#### 3.5.2. Fourier Transform Infrared (FTIR) Spectroscopy

**Figure 8:**
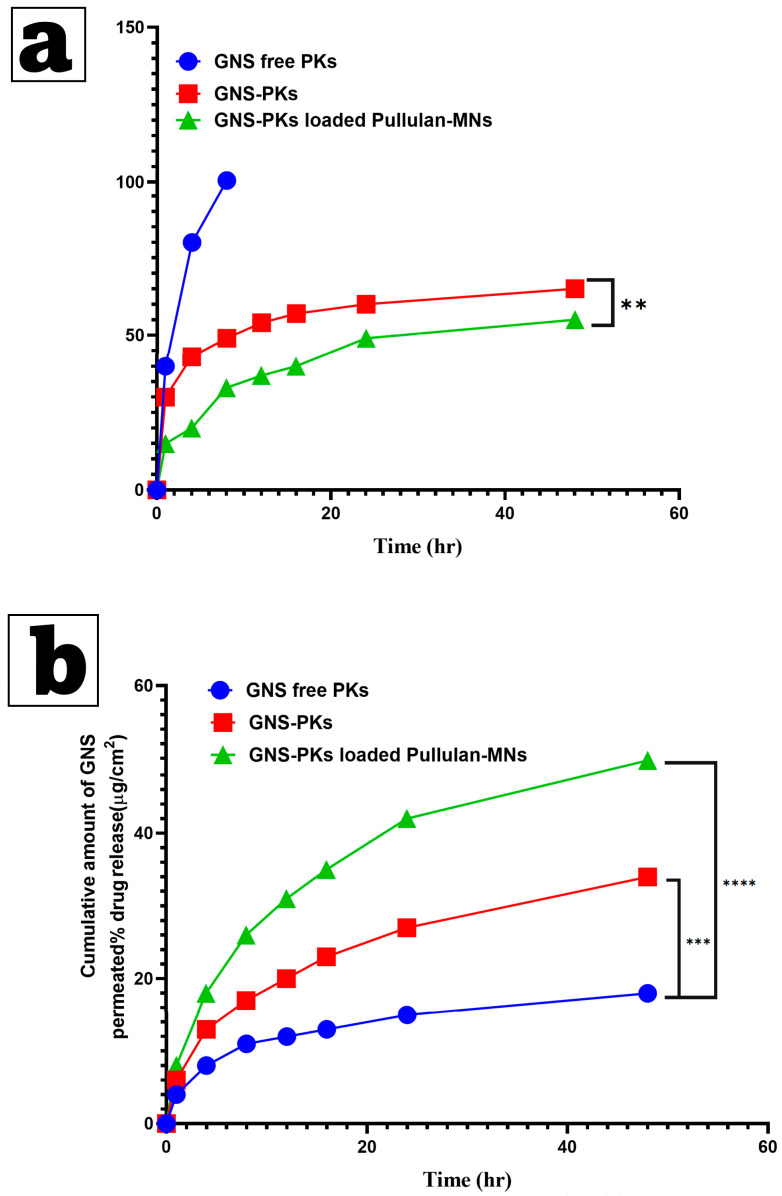
(**a**) In vitro drug release of GNS from GNS-PKs and GNS-PKs-loaded Pullulan MNs, and (**b**) Ex vivo permeation study for different formulations. Data were expressed as Mean ± SD, *n* = 3. ** *p* < 0.01, *** *p* < 0.001, and **** *p* < 0.0001 versus the control group.

Figure 8 Show the Blank/MNs spectrum; a broad O–H stretching band across 3200–3400 cm^−1^, characteristic of hydrophilic polymers (e.g., PVA, PVP), C–H stretching around 2900 cm^−1^, C=O stretching near 1650–1730 cm^−1^, reflecting the polymeric matrix. Intense fingerprint-region peaks at 1000–1200 cm^−1^, associated with polymer backbone vibrations. This confirms the structural fingerprint of the microneedle-forming polymers. The distinctive GNS peaks (e.g., carbonyl and aromatic bands) were highly attenuated or absent, due to complete embedding of the nano-pumpkisomes within the hydrophilic microneedle network. Minor shifting or broadening in the 1500–1700 cm^−1^ region is attributed to physical interactions (hydrogen bonding and van der Waals forces) between the vesicles and the polymer chains. The disappearance or reduction of GSN characteristic peaks in the GSNUFs-MNs spectrum confirms successful incorporation of drug-loaded vesicles into the microneedle matrix, with no chemical degradation or interaction between GSN and the polymers. The vesicular system remains structurally intact within the MNs, and the Drug is protected within the nano-lipid vesicles and further embedded in the MN polymer network. GNS fingerprint peaks are clearly visible only in the pure drug spectrum; their attenuation in GNS-PKs indicates efficient encapsulation within pumpkisomes. Their complete masking in GNS-PKs-MNs confirms the successful entrapment of the nanovesicles within the microneedles. No new peaks appeared, demonstrating that the formulation components interact physically rather than chemically. The preservation of characteristic lipid and polymer peaks confirms the structural compatibility and stability of all components [18,124].

#### 3.5.3. Differential Scanning Calorimetry (DSC)

Figure 5b DSC thermograms were used to evaluate the thermal behavior of blank microneedles (Blank/MNs) and drug-loaded microneedles (GNS-PKs/MNs). Blank/MNs exhibited a broad, stable temperature profile with no sharp transitions, confirming the amorphous polymeric composition of the microneedle matrix. The smooth profile indicates that pullulan forms an amorphous network without crystalline transitions at the observed temperatures. With no melting peak for GNs, no significant thermal event for lipid vesicles, and a smooth, polymer-dominated thermogram, (GNS-PKs/MNs) showed a DSC curve similar to that of Blank/MNs. The absence of GNs melting peaks confirms the drug’s amorphization within the microneedle matrix. The similarity between Blank/MNs and (GNS-PKs/MNs) suggests that no chemical interactions alter the polymer’s thermal behavior. Nano-pumpkisomes are physically embedded and stabilized within hydrophilic polymers. The preservation of microneedle thermal structure demonstrates the compatibility and stability of drug-loaded vesicles with MN materials.

#### 3.5.4. Drug Release Studies

##### In Vitro Drug Release Study and Kinetics Analysis

In vitro release profiles of GNS-free PKs, GNS-PKs, and GNS-PKs-Pullulan MNs exhibited distinct patterns that reflected the structural properties and diffusion barriers of each formulation (Figure 8a). Free GNS released over 80% of the medication in the first 4 h, with the remaining 20% released after 8 h. The non-encapsulated drug disintegrates immediately in the release medium, allowing unrestricted diffusion over the dialysis membrane. This formulation cannot achieve a steady-state flux because it lacks a physical or polymeric barrier that would enable sustained release. GNS-PKs nanovesicles are released gradually. After 1 h, 30% of the GNS was released; after 4 h, 43%; after 24 h, 60%; and after 48 h, 65%. Encapsulation of genistein in the pumpkisome nanostructure creates a diffusion-resistant matrix that prevents drug escape, which explains the consistent trend. The nanovesicle structure regulates drug release from the PK matrix, yielding a steady-state flow rate of 0.208 µg/cm^2^/hr in the later stages of the study. Nanovesicle encapsulation regulates release kinetics and prevents premature drug dumping, setting it apart from free GNS. The most extended and controlled release profile was observed with GNS-PKs-loaded pullulan microneedles. Only 15% of the drug was released in the first hour, 49% after 24 h, and 55% after 48 h. Release is slower due to the pumpkisomes nanoparticle matrix and microneedle pullulan polymer network’s sequential diffusion barriers. Once the microneedles fracture, nanoparticles remain suspended in the hydrated pullulan gel, thereby restricting drug diffusion. The release profile exhibits a smoother, more prolonged profile than that of the nanoparticle suspension alone, with a higher steady-state flow rate of 0.250 µg/cm^2^/h. Collectively, these data confirm that the system operates as a dual-compartment, sequential-release platform, in which GNS and P.O-derived fatty acids are first released from the pumpkisome bilayer in a diffusion-controlled manner and then further modulated by diffusion through the dissolving pullulan microneedle matrix. This dual-barrier technology effectively mitigates the initial burst effect of free drug while facilitating continuous medication delivery.

Table 6 presents the kinetic modeling of genistein release; the coefficients were consistently low, indicating that neither the zero-order nor the first-order model adequately fit the results. The highest R^2^ values for the Higuchi model were 0.982 for free GNS, 0.879 for GNS-PKs, and 0.941 for GNS-PKs MNs, indicating that Fickian diffusion is the primary mechanism for release. The nanovesicle matrix governs the release of GNS-PKs during drug transport, whereas free GNS can diffuse through the membrane. Nanoparticles diffuse through the hydrated pullulan matrix in the microneedle device, slowing and prolonging release. Free GNS releases immediately, pumpkisomes control diffusion, and GNS-PKs on pullulan microneedles offer the most sustained, diffusion-driven release. The best fit with the Higuchi model across all systems indicates that Fickian diffusion governs genistein release under the experimental conditions. The prolonged release of the GNS-PKs pullulan microneedle platform suggests it could extend therapeutic availability following transdermal application.

##### Ex Vivo Drug Permeation

Figure 8 Ex vivo permeation through excised skin showed a markedly different trend than the in vitro release study. The highest permeation was observed in GNS-PKs loaded with MNs, followed by GNS-PKs; free GNS exhibited the lowest permeation. The use of MN-assisted delivery led to a notable enhancement in cumulative permeation, achieving approximately 45–50 µg/cm^2^. This result was significantly greater than that for both GNS-PKs and free GNS (*p* < 0.0001). This improvement results from the use of microneedles to bypass the stratum corneum, thereby facilitating the direct delivery of nanoparticles into the viable epidermal layers. The GNS-PKs formulation, excluding MNs, demonstrated an intermediate level of permeation, approximately 25–30 µg/cm^2^. The nanoscale dimensions limit skin penetration and slow diffusion across the skin barrier; however, absorption remains constrained in the absence of physical enhancement methods. Free GNS demonstrated the lowest permeation levels, ranging from approximately 12 to 15 µg/cm^2^. This observation indicates its limited solubility, reduced skin permeability, and absence of support from nanocarriers or microneedles. The hydrophobic nature of GNS limits its passage through the skin’s hydrophilic pathways [125]. MN-loaded nanoparticles provide the greatest permeation enhancement, achieving the highest cumulative drug levels by creating microchannels through the skin, rapid dissolution of pullulan MNs, and direct deposition of nanoparticles into deeper layers, thereby improving diffusion gradients. Statistical significance (*p* < 0.0001) confirms that MN-assisted delivery provides a superior transdermal drug delivery approach. Thus, GNS-PK-loaded pullulan microneedles represent the most efficient system, combining controlled release and enhanced skin permeation into a single platform.

### 3.6. In Vitro Cell Culture Studies

**Figure 9:**
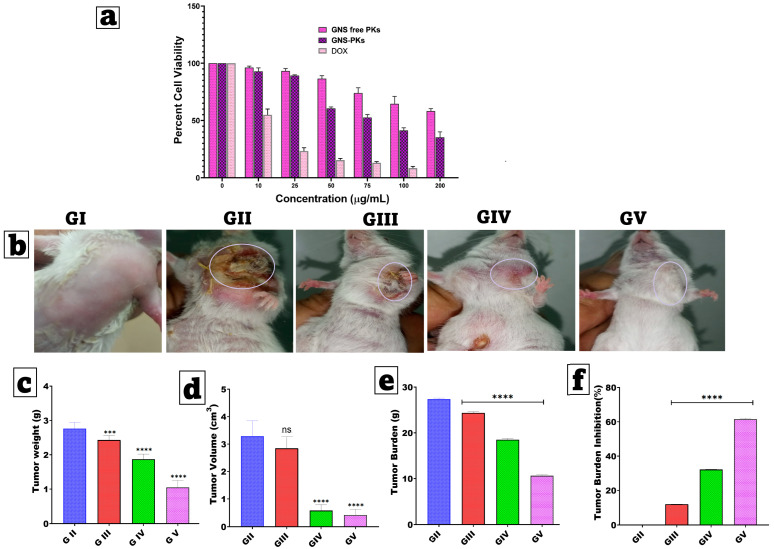
Effects of different formulation of genistein in model induced by Ehrlich in various groups on (**a**) Cell viability, evaluated by MTT cell proliferation assay, of MCF-7 cells after incubation for 24 h, (**b**) Morphology of skin and tumor, (**c**) tumor weight, (**d**) volume, (**e**) burden, and tumor (**f**) burden inhibition % of female Swiss albino mice. Data were expressed as Mean ± SD, *n* = 3. *** *p* < 0.001, **** *p* < 0.0001, and ns (not significant) versus the control group.

Figure 9a Presents cytotoxicity evaluations of GNS formulations compared to DOX, revealing notable variations in their impact on cell viability across the examined concentration range. The figure shows that both GNS-free PKs and GNS-PKs demonstrated a concentration-dependent decline in percent cell viability, suggesting that higher doses of genistein correspondingly diminished cellular proliferation. Treatment with GNS-PKs led to a concentration-dependent decrease in MCF-7 cell viability, exhibiting significantly greater cytotoxicity than GNS-free PKs (*p* < 0.05). DOX, serving as positive control, demonstrated the highest potency, reducing viability to approximately 55% at 10 µg/mL and to less than 10% at 100 µg/mL or higher. In contrast, GNS-PKs exhibited significantly higher cell viability across all doses, indicating an enhanced safety profile for the developed formulation compared with the clinical chemotherapeutic DOX. This suggests that the fundamental cellular interaction with the pumpkisomes carrier may intrinsically reduce spontaneous proliferation, likely due to enhanced nanoparticle uptake or mild metabolic stress induced by the nanocarrier system [1,86]. With the drug concentration increased to 50–100 µg/mL, both formulations showed a gradual decrease in cell viability; however, GNS-PKs showed a greater reduction at each dose. At a concentration of 100 µg/mL, free GNS exhibited a viability of approximately 41%, while GNS-PKs demonstrated a more pronounced reduction in viability, dropping to around 65%. The enhanced cytotoxic effect results from the nanoencapsulation of genistein within pumpkisomes. This process enhances cellular penetration, protects the drug from degradation, and increases intracellular accumulation, thereby fostering a more robust antiproliferative response. At the highest concentration tested (200 µg/mL), GNS-PKs exhibited the most remarkable cytotoxicity, reducing cell viability to approximately 35%, compared with ~58% for free GNS. This further supports the notion that the nanoparticle-mediated delivery considerably enhances the bioavailability and potency of genistein within cells. The results indicate that GNS-PKs are the more effective cytotoxic treatment, producing greater reductions in cell viability across all concentrations. The IC_50_ of the optimized GNS-PKs (3.5 µg/mL) is markedly lower than free genistein, which typically ranges between 12–40 µg/mL in MCF-7 cells, and is superior to most reported nano-genistein systems (4–10 µg/mL), indicating enhanced potency attributable to P.O.-based vesicular delivery. GNS-free PKs significantly decreased MCF-7 cell viability relative to untreated controls in a concentration-dependent manner, demonstrating that the pumpkin-oil-based vesicular carrier has intrinsic antiproliferative effects. This observation reinforces our classification of pumpkisomes as a bioactive co-therapeutic component, rather than an inert vehicle. It aligns with the established anticancer activity of OA and LA in breast cancer models. The improved activity can be attributed to the nanoscale formulation, which facilitates superior cellular internalization and sustained intracellular release of genistein relative to the free drug. These findings highlight the therapeutic advantage of pumpkisomes delivery for enhancing the breast anticancer potential of genistein.

### 3.7. In Vivo Study

#### 3.7.1. Estimation of the Morphology of the Skin and Tumor and Body Weight

Figure 9b shows the morphological Changes in Skin and Tumor; GI (negative control) has skin that appears perfectly normal, with a smooth texture, unbroken epidermis, and no lumps, ulceration, or inflammation, indicating the absence of tumor induction. GII (Positive Ehrlich Tumor Control) exhibited significant pathology, such as widespread skin ulceration, necrotic crusts, yellowish inflammatory exudate, and prominent edema and erythema. These characteristics correspond to aggressive, rapidly proliferating, untreated Ehrlich tumors with significant tissue damage. GIII (GNS Gel) demonstrated partial improvement. Ulceration was minimized, and scabbing was confined; nonetheless, the tumor mass remained visible despite substantial skin disturbance, indicating poor tumor progression inhibition. GIV (GNS-PKs Gel) resulted in evident morphological recovery, including smaller, well-defined tumors, intact skin, minimal discoloration, and reduced edema. The absence of necrotic crust indicates improved tissue viability and enhanced therapeutic efficacy. GV (GNS-PKs Microneedles) demonstrated the best therapeutic outcomes. Animals were observed for local inflammatory responses, including erythema, edema, and ulceration, during the treatment period. No visible skin irritation or ulceration was noted, and histopathological examination of skin sections near the MN application site revealed no inflammatory infiltration linked to the gel or polymeric matrix. Tumors were significantly smaller and better defined, with intact skin, no ulceration, limited edema, and no necrotic areas, indicating significant tumor inhibition and superior drug delivery by microneedles. Figure 9c showed that the highest tumor weights were consistently observed in Group II (untreated), confirming the aggressive growth of SEC tumors in the absence of intervention. Treatment with pure genistein (Group III) resulted in a modest yet statistically significant decrease in tumor weight, whereas GNS-PKs gel (Group IV) exhibited an additional reduction. The most significant decrease was observed in the recorticated skin, with minimal discoloration in Group V (GNS-PKs MNs), in which tumors were either very small or nearly undetectable.

This study investigated the effects of several genistein formulations on body weight in female Swiss albino mice. As shown in Table 7, group I, the negative control group, exhibited a notable increase in body weight from 25 ± 0.12 g to 52 ± 0.23 g, signifying robust growth and the absence of disease or treatment-related adverse effects. The disease model group (Group II) exhibited a negligible increase in body weight (24 ± 0.19 g to 26 ± 0.19 g) following induction of the Ehrlich tumor without any therapeutic intervention. This minimal rise aligns with the effects of tumor burden, such as cachexia, inadequate nutrition, and cancer-related catabolism. Mice in Group III, which received genistein-loaded HPMC gel, exhibited a significant increase in body weight, rising from 25 ± 0.23 g to 35 ± 0.32 g. Topical application of genistein in an HPMC-based gel may partially mitigate the metabolic consequences of tumor growth. Nevertheless, Group IV animals administered genistein encapsulated in pumpkin-seed oil vesicles (GNS-PKs) within the HPMC gel matrix exhibited greater improvement. The nanocarrier technique enhanced genistein delivery, resulting in a rise in body weight from 25 ± 0.12 g to 40 ± 0.15 g, likely attributable to improved bioavailability and sustained release, which mitigated tumor-induced weight loss. Group V exhibited optimal results with GNS-PKs administered with Pullulan microneedle patches. Body weight increased in these animals to a level comparable to that of healthy controls, rising from 23 ± 0.23 g to 49 ± 0.29 g. The notable restoration of body weight indicates that microneedle transdermal delivery may enhance systemic absorption, circumvent gastrointestinal degradation, and facilitate a gradual release of the active compound. The route of administration of genistein is crucial to its therapeutic effectiveness. Genistein alone is beneficial; however, its efficacy in mitigating the cachectic effects of Ehrlich tumors in mice is significantly enhanced when incorporated with advanced delivery systems, such as transdermal microneedle-administered pumpkin-seed oil vesicles. This indicates that nanocarrier and microneedle drug delivery systems can improve the efficacy of cancer therapy, patient health, and overall quality of life.

#### 3.7.2. Estimation of Tumor Parameters

As shown in Figure 9d, the parallel results for weight showed that tumor volumes were substantially higher in untreated mice and lowest in the MN-treated group, with intermediate values for other treatment arms. This illustrates the greater impact of formulation and delivery methods on both tumor size and tumor progression.

Figure 9e,f showed the tumor Burden and Tumor Burden Inhibition (%). Defined as the cumulative tumor mass for each group, the tumor burden mirrored individual tumor measurements, being highest in untreated controls and lowest in the GNS-PKs MNs group. This statistic, expressed as the percentage decrease in tumor burden relative to the untreated group, provides a straightforward, quantitative measure of therapeutic efficacy. Group III (Pure GNS-HPMC gel) exhibited modest inhibition (~15–20%), Group IV (GNS-PKs) displayed greater inhibition (~35–40%), whereas Group V (GNS-PKs MNs) achieved the most significant inhibition (>60%), indicating a substantial therapeutic advantage. The percentage of tumor burden inhibition is a standardized metric for comparing treatment outcomes across groups. The notable increase in inhibition from Group III (Pure GNS) to Group IV (GNS-PKs) to Group V (GNS-PKs MNs) illustrates the cumulative benefits of each formulation improvement; Unadulterated genistein: Partial effect limited by delivery. GNS-PKs demonstrated enhanced efficacy resulting from optimized administration, prolonged release, and increased bioavailability. GNS-PKs Pullulan MNs exhibited optimal inhibitory effects, enhancing local tumor therapy through direct, effective, and minimally invasive delivery. Pure drugs, nano-formulated drugs, and nano-formulated drugs delivered via microneedles inhibit tumor proliferation. This may arise from enhanced drug absorption, tumor tissue infiltration, localized immune activation, or pharmacodynamic factors. Untreated tumor-bearing mice demonstrated accelerated tumor growth, significant skin damage, and marked weight loss, resembling SEC. Genistein alone provided minimal protection due to insufficient uptake and absorption. The GNS-PKs nanoformulation in gel form enhanced cellular absorption and prolonged release, resulting in reduced tumor growth and pathogenesis. The GNS-PKs-loaded Pullulan MNs demonstrated the highest efficacy, significantly reducing tumor growth and returning animal health to baseline levels. This delivery method has the potential to enhance tumor drug concentration, circumvent first-pass metabolism, and reduce systemic toxicity. The study highlights the necessity for novel GNS delivery methods. Nanovesicle pumpkisomes encapsulation and microneedle delivery enhance solubility and metabolic processes. This dual-platform strategy enhances efficacy while minimizing systemic exposure and toxicity. These approaches are crucial for translational cancer therapy, particularly for locally advanced or cutaneously involved malignancies. The tumor-suppressing, animal longevity, and physiological benefits of GNS-PKs Pullulan MNs indicate their therapeutic promise for breast cancer and other solid tumors.

#### 3.7.3. Assessment of Inflammatory Biomarkers

**Figure 10:**
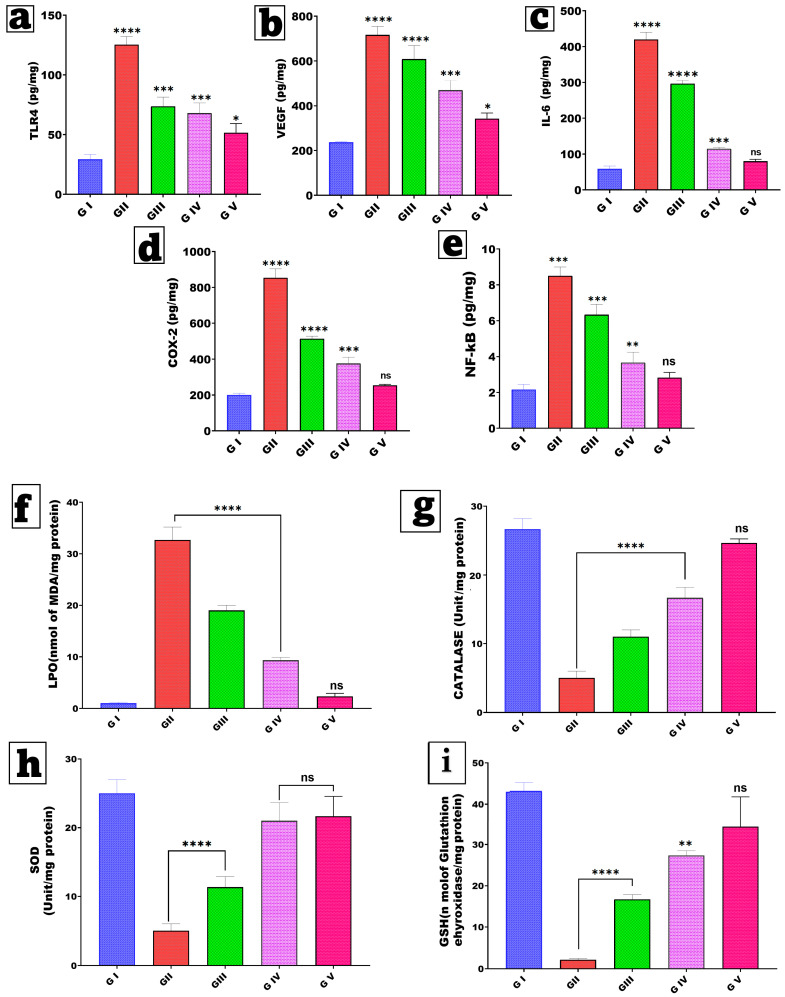
Effects of different treatments on inflammatory biomarkers measured by ELISA: (**a**) TLR4, (**b**) VEGF, (**c**) IL-6, (**d**) COX-2, and (**e**) NF-kB. (**f**) Lipid peroxidation (LPO, expressed as MDA levels). (**g**–**i**) Antioxidant activities: (**g**) catalase, (**h**) superoxide dismutase (SOD), and (**i**) glutathione (GSH). Data are expressed as mean ± SD (*n* = 10). * *p* < 0.05, ** *p* < 0.01, *** *p* < 0.001, **** *p* < 0.0001, and ns (not significant) versus the control group. Abbreviations: Group I (normal group, negative control), Group II (disease model group, positive control), Group III (treated with GNS loaded with gel containing 2% (*w*/*w*) HPMC), Group IV (treated with GNS-PKs loaded with gel containing 2% (*w*/*w*) HPMC), Group V (GNS-PKs-loaded Pullulan MNs). Data were expressed as Mean ± SD, *n* = 3.

Figure 10a showed the inflammatory marker TLR4; Group II had significantly higher TLR4 levels, indicating strong innate immune activation and chronic inflammation driven by tumor presence. Groups III–V showed that all treatments reduced TLR4 compared with Group II, but the reduction was most pronounced in Group V (GNS-PKs MNs), approaching normal levels. TLR4 upregulation is associated with tumor progression and immune evasion; its reduction reflects attenuation of the tumor’s pro-inflammatory environment, particularly with advanced drug delivery [126].

Figure 10b showed the inflammatory markers VEGF; Group II: Markedly elevated VEGF, reflecting robust angiogenesis (formation of new blood vessels feeding tumor growth). Groups III–V showed a progressive reduction, with Group V showing the lowest VEGF levels relative to the negative control, indicating the most potent inhibition of tumor angiogenesis. VEGF inhibition reduces tumor blood supply and metastasis risk, supporting the anti-angiogenic efficacy of GNS-PKs, especially via MNs [127].

Figure 10c showed the inflammatory marker IL-6; Group II had high IL-6, a cytokine strongly associated with inflammation, cancer proliferation, and resistance to therapy. Groups III–V show significantly suppressed IL-6; Group V almost normalizes IL-6, highlighting a potent anti-inflammatory effect. Lower IL-6 levels reduce tumor proliferation and resistance mechanisms, underscoring the immunomodulatory role of the tested formulations [128].

Figure 10d showed the inflammatory marker COX-2. Group II showed extreme elevation of COX-2, consistent with inflammation and tumorigenesis. Groups III–V showed a marked reduction with treatment, and in Group V, levels were comparable to those in the control. COX-2 is a classic marker of chronic inflammation and cancer; its reduction suggests successful suppression of pro-tumor inflammatory cascades [129,130].

Figure 10e showed the inflammatory marker NF-κB; Group II showed significant improvement, suggesting activation of chronic inflammatory signaling and tumor-survival pathways. Groups III–V are treatment-reduced NF-κB, with Group V again approaching normal levels. Inhibiting NF-κB disrupts survival signals and tumor-promoting inflammation. Inhibiting NF-κB impairs survival signals and promotes tumor inflammation. All inflammatory biomarkers were highest in tumor-bearing mice that had not been treated, and therapy significantly reduced them, with GNS-PKs MNs showing the most complete reduction. This demonstrates the improved formulation’s capacity to modify the tumor microenvironment, inhibit pro-tumor inflammation, and disrupt signaling pathways critical to tumor maintenance and growth [130,131].

#### 3.7.4. Assay of Oxidative Stress Parameters

Figure 10f showed a significant reduction in lipid peroxidation in the treated groups, particularly in GNS-PKs Pullulan MNs, indicating that the employed formulation methods provide substantial protection against tumor-induced oxidative damage. These treatments inhibit cancer progression by reducing excessive lipid peroxidation (LPO) while simultaneously enhancing the host’s overall health and resilience, thereby establishing this approach as an effective anti-cancer and cytoprotective therapy.

#### 3.7.5. Enzymatic Antioxidant Parameters

Figure 10g showed catalase; group II had severely decreased catalase activity, indicating loss of natural antioxidant defenses. Groups III–V are progressive restoration, with Group V fully restoring catalase activity. Restoration of catalase suggests enhanced antioxidant protection against reactive oxygen species (ROS) induced by cancer.

Figure 10h showed SOD; group II is drastically reduced, further demonstrating compromised antioxidant defense. Groups III–V show significant recovery, especially in Groups IV and V, where SOD approaches or even surpasses normal levels. Replenished SOD effectively neutralizes superoxide radicals, limiting ROS-driven tumor progression. The marked decrease in lipid peroxidation across treated groups, and especially in GNS-PKs Pullulan MNs, demonstrates that your formulation strategies provide robust protection against tumor-induced oxidative damage. By curbing excessive LPO, these treatments not only inhibit cancer progression but also enhance the host’s overall health and resilience, positioning this approach as a potent anti-cancer and cytoprotective strategy [1].

#### 3.7.6. Non-Enzymatic Antioxidant Parameters

Figure 10i showed GSH; Group II: Markedly reduced glutathione, a master non-enzymatic antioxidant, due to tumor-induced oxidative stress. Groups III–V; stepwise restoration, with Group V returning to near-control levels. Higher GSH reflects successful restoration of cellular redox homeostasis. Untreated tumors (Group II) exhibit a classic profile of chronic inflammation (high TLR4, IL-6, COX-2, VEGF, NF-κB), high oxidative damage (LPO), and suppressed antioxidant defense (CAT, SOD, GSH). Genistein (especially in microneedle forms) powerfully reverses this profile, as evidenced by suppression of key inflammatory mediators (reduced TLR4, IL-6, VEGF, COX-2, NF-κB), reduction of oxidative damage (lower LPO/MDA), and restoration of antioxidant defenses (higher CAT, SOD, GSH). Efficacy Hierarchy; GNS-PKs-loaded Pullulan MNs (Group V) > GNS-PKs Gel (Group IV) > Pure genistein Gel (Group III) > Untreated.

The most advanced formulation (GNS-PKs MNs) achieved nearly complete normalization of all markers, indicating profound therapeutic and chemo preventive effects. These results demonstrate that not only does genistein act as a direct anti-tumor and anti-inflammatory agent, but its nanoencapsulation (pumpkisomes) and transdermal delivery (MNs) dramatically amplify its effects, providing potent inhibition of tumor-promoting inflammation and angiogenesis, substantial mitigation of oxidative stress and restoration of antioxidant balance, and superior overall efficacy against tumor growth and microenvironmental dysregulation. These GNS-PKs-loaded Pullulan MNs are an up-and-coming, multi-targeted platform for effective breast cancer therapy, targeting both tumor cells and their inflammatory/oxidative microenvironment [1,6].

#### 3.7.7. Liver Function Tests (LFTs), Kidney Function Tests, and Sex Hormone Assessment

Liver enzymes and bilirubin serve as sensitive indicators of hepatic health and potential toxicity. Table 8 showed that the mice in Group II have a dramatic elevation in total bilirubin, SGOT, SGPT, and ALP, classic signs of significant liver injury, likely due to both tumor progression and paraneoplastic effects. High SGOT, SGPT, and ALP indicate hepatocellular damage and/or cholestasis, while elevated bilirubin indicates compromised hepatic clearance. These indicators are considerably lower in treated Groups (III–V) than in Group II, indicating a dose-dependent impact on liver protection. Group III (GNS) LFTs were substantially lower but still above normal. Group IV (GNS-PKs) has better normalization of all indicators, suggesting sustained release and focused delivery increase hepatic protection. Group V almost entirely reversed liver damage with near-normal LFTs. This advanced therapy was effective and safe because most measures (e.g., total bilirubin, SGOT, SGPT, ALP) approached healthy control levels and differences were not statistically significant (“ns”). Cancer development and several chemotherapies cause hepatotoxicity by inflammation, ROS, and direct cytotoxicity. Genistein, particularly when nanoencapsulated and transdermally administered, can attenuate hepatic oxidative and inflammatory insults without undergoing first-pass hepatic metabolism or causing systemic excess.

Testing kidney function (urea, uric acid, creatinine). In group II, these markers were highly elevated, indicating renal dysfunction or injury due to tumor-related cachexia, metabolic stress, or tumor microenvironment nephrotoxicants. Group III had partial correction, but values remained elevated; Group IV had additional reduction, with kidney indicators approaching normal; and Group V had most kidney function markers (urea, uric acid, creatinine) normalized or showed only slight deviations from healthy controls. Renal function is crucial in cancer treatment to prevent problems and aid recovery. These results suggest that GNS-PKs, notably in MNs, protect the kidneys by reducing oxidative stress and systemic inflammation [132,133].

Sex Hormone Assessment (Progesterone and Estrogen); in which Group II has both hormones (especially estrogen) significantly elevated compared to normal mice, which may reflect tumor-driven dysregulation of endocrine function, especially in hormone-sensitive cancers like breast cancer. Group III: the hormone levels decreased toward normal but remained higher than those of healthy controls. Group IV: further reduction, with improved regulation of hormone levels. Group V had hormone levels that were nearly normalized, with no significant difference relative to controls. Uncontrolled tumor growth often perturbs systemic hormone balance [134]. Restoration of hormone levels by your treatments suggests not only antitumor efficacy but also a return to physiological homeostasis, which is critical for reducing the risk of cancer progression and improving quality of life.

#### 3.7.8. Lipid Profile

Table 8 showed cholesterol, triglycerides, and HDL in Group II (Untreated Tumor), where total cholesterol and triglycerides were severely elevated, a hallmark of cancer-induced dyslipidemia, which can promote tumor growth, invasion, and metastasis. HDL cholesterol: sharply decreased, reflecting impaired lipid metabolism and antioxidant transport low HDL is associated with worse cancer prognosis. Group III has partial improvement; cholesterol and triglycerides decreased, and HDL rose slightly. Group IV has marked normalization, with values close to those of healthy mice. Group V has almost completely restored its normal lipid profile, with HDL levels much higher than they were before. Cancer changes how lipids are broken down and used to speed up cell growth and change how the immune system works. The capacity of GNS-PKs, particularly when administered through Pullulan MNs, to correct lipid profiles not only validates tumor suppression but may also mitigate the risk of cancer-related cardiovascular disease and metabolic disorders. Mice with tumors that weren’t treated displayed typical paraneoplastic syndromes, such as severe liver and kidney damage, hormone abnormalities, and dyslipidemia. Genistein therapies, especially in advanced forms such as nanopumpkisomes and microneedles, significantly improved physiological parameters [1,135]. They restored liver and kidney function, corrected hormone imbalances, and normalized the lipid profile. GNS-PKs Pullulan MNs (Group V) were always the best, bringing all markers back to normal almost entirely. This shows that it is both very effective against cancer and very safe (it doesn’t hurt organs). These results strongly suggest that enhanced genistein formulations (GNS-PKs, especially when administered using Pullulan MNs) not only stop tumors from growing but also guard against the metabolic problems and organ damage that often come with cancer and its treatments. Although MDA was quantified as an index of lipid peroxidation, no significant elevation was observed in SEC-bearing mice, likely due to the strong induction of endogenous antioxidant enzymes (SOD, CAT, GSH), which may have attenuated downstream lipid-damage markers, as shown in Figure 10. Our results go along with what has been already reported that there is no direct link between antioxidant biomarkers (SOD/CAT/GSH) and MDA especially in diseased status such as cancer. MDA is increased in pathological conditions such as diabetes indicating the widespread of lipid damage [6,8,9]. This dual benefit supports the potential clinical utility of your system in safe, effective breast cancer management.

### 3.8. Histopathologic Analysis

**Figure 11:**
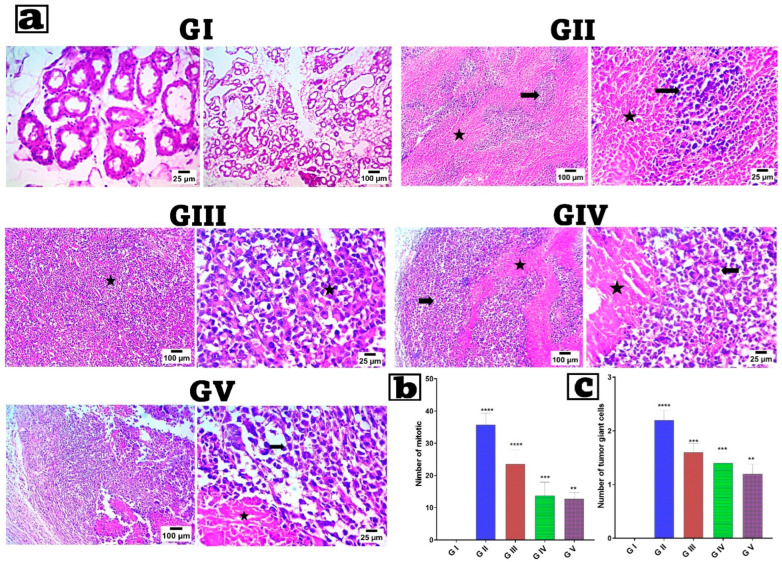
(**a**) Representative hematoxylin and eosin (H&E)–stained photomicrographs of dissected solid Ehrlich carcinoma (SEC) tissue sections from different experimental groups, imaged at ×100 and ×25 magnifications. Sections illustrate aggregates of neoplastic cells with mitotic figures (black arrows) and areas of necrosis (stars). The effects of genistein formulations on tumor histopathology are shown across the groups: GI (healthy negative control), GII (SEC model, untreated), GIII (genistein gel with 2% *w*/*w* HPMC), GIV (genistein–pumpkisomes nanovesicles in gel with 2% *w*/*w* HPMC), and GV (genistein–pumpkisomes nanovesicles in G/HA/CMC microneedles); (**b**) Quantitative analysis of the average number of mitotic cells, and (**c**) Quantitative analysis of the average number of giant tumor cells. Data are presented as mean ± SD (*n* = 10). ** *p* < 0.01, *** *p* < 0.001, **** *p* < 0.0001 vs. control.

The solid Ehrlich carcinoma (SEC) model is widely used in preclinical research to assess anticancer therapies. Its consistent tumorigenic potential and capacity to replicate critical features of human breast cancer, such as rapid proliferation, necrosis, and cellular atypia, contribute to its prominence in the field. This study revealed notable histopathological differences among experimental groups, as indicated by H&E staining (Figure 11a). The negative control group (GI) exhibited normal tissue architecture, characterized by well-organized cellular and glandular structures, with no evidence of neoplastic transformation, mitosis, or necrosis. The SEC model group (GII) exhibited substantial aggregates of neoplastic cells, marked by numerous mitotic figures (black arrows) and extensive necrotic areas (star). Significant mitotic activity and the presence of giant tumor cells indicate an aggressive tumor biology, reflecting uncontrolled cellular proliferation and accelerated tumor growth. The necrosis observed in this group is likely attributable to differences in tumor growth and blood flow, resulting in hypoxia and cell death. Compared with the untreated SEC group, the groups administered genistein, either in conventional gel (GIII) or in nanoformulations (GIV, GV), exhibited notable improvements in morphological characteristics. The data presented in Figure 11b,c indicate that all treated groups showed a significant reduction in both mitotic and large tumor cells. Furthermore, GNS-PKs reduced necrotic areas, with the most critical effect observed in the GV group that received microneedle administration. The use of GNS-PKs–Pullulan -MNs (GV), significantly improves intratumoral drug penetration, facilitates sustained release, and enhances cellular uptake, thereby boosting anti-tumor activity. Histological results demonstrate that genistein and its optimized formulations disrupt the cell cycle, reduce neoplastic proliferation, and promote tumor cell apoptosis. Genistein treatment suppresses tyrosine kinases and modulates apoptotic pathways, leading to reduced cell proliferation and increased apoptosis, thereby accounting for the noted decrease in mitotic and large cells. Nanoformulations and transdermal delivery systems have improved therapeutic efficacy, demonstrating that innovative drug delivery methods can address the limitations of traditional treatments, particularly regarding bioavailability and rapid elimination [136].

### 3.9. Immunohistochemical (IHC) Examination of Epidermal Growth Factor Receptor (EGFR)

The epidermal growth factor receptor (EGFR) is crucial in the progression, proliferation, and survival of breast cancer tumors. Overexpression of EGFR correlates with higher tumor grade, increased mitotic index, resistance to apoptosis, and a generally unfavorable prognosis. The current study’s immunohistochemical analysis revealed a significant increase in EGFR expression in the untreated tumor-bearing group (GII), corroborating the aggressive neoplastic phenotype typical of the EAC model [137].

**Figure 12:**
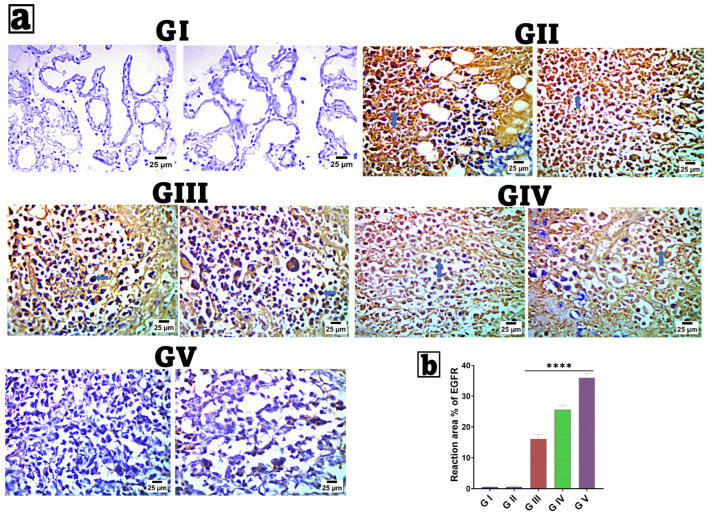
(**a**) Immunohistochemical photomicrographs of lung sections stained for Epidermal Growth Factor Receptor (EGFR) expression in different groups. Severe positive EGFR expression is indicated in neoplastic cells (arrows). Group assignments: GI (healthy negative control), GII (SEC model, untreated), GIII (treated with genistein gel containing 2% *w*/*w* HPMC), GIV (treated with genistein–pumpkisomes nanovesicles in gel with 2% *w*/*w* HPMC), and GV (treated with genistein–pumpkisomes nanovesicles incorporated in G/HA/CMC microneedles). Scale bar = 25 µm. (**b**) Quantitative analysis of EGFR immunoreactivity, presented as reaction area percentage (%). Data are expressed as mean ± SD (*n* = 10). **** *p* < 0.0001 versus the negative control group.

As shown in Figure 12, the negative control group (GI) showed no EGFR immunoreactivity, indicating the preservation of standard tissue architecture. The model group (GII) demonstrated notable EGFR positivity, aligning with existing literature that links EGFR overexpression to uncontrolled cellular proliferation and tumorigenesis in breast cancer. The treated groups (GIII, GIV, and GV) exhibited a notable decrease in EGFR expression after genistein treatment, particularly when delivered via advanced nanovesicle formulations (pumpkisomes) and microneedle arrays, relative to the untreated group. Blocking EGFR-mediated signaling pathways reduces tumor cell growth and may promote cell death. The most significant benefit was observed in group GV (genistein–pumpkin microneedle formulation), which had EGFR levels comparable to those of the healthy control group. EGFR suppression in vivo is consistent with genistein’s known modulation of estrogen receptor signaling, inhibition of tyrosine kinase phosphorylation, and induction of apoptosis via Bcl-2/Bax and caspase activation, supporting a coordinated multimodal mechanism rather than a single-node effect. Our findings for our new formulation of genistein (genistein–pumpkin microneedle formulation) goes along with that has been already reported by Tanjak et al. [130]. The synergistic effect of nanovesicle-mediated delivery with transdermal administration may increase tumor-site drug concentrations, enhance cellular uptake, and overcome the constraints of standard delivery methods.

## 4. Conclusions

This study demonstrates that incorporating genistein-loaded pumpkin-derived nanovesicles into pullulan-based disintegrating microneedle patches provides a novel and practical approach for targeted breast cancer treatment. The optimized nanovesicle formulation exhibited high entrapment efficiency, nanoscale particle size, and considerable stability, while the microneedle arrays enabled effective skin penetration and prolonged drug release. In vitro and in vivo studies demonstrated that this dual-delivery system markedly inhibited tumor growth, reduced the proportion of mitotic and giant tumor cells, and suppressed EGFR expression in the solid Ehrlich carcinoma model. The treatment also increased antioxidant activity and reduced inflammatory biomarkers, indicating minimal systemic toxicity. The findings underscore the potential of this dual-delivery platform as a minimally invasive, effective method for breast cancer management. This study provides preclinical proof-of-concept evidence that GNS-PK-loaded microneedles improve anticancer and antioxidant efficacy in the SEC model. While encouraging, the results are limited to a single tumor model and short-term exposure; more study involving pharmacokinetics, toxicity profiles, HER2-specific breast cancer models, and biodistribution studies is required before any translational consideration.

## Figures and Tables

**Table 1:** I-Optimal factorial design utilized for optimizing GNS-PKs formulations.

Factors (Independent Variables)	Design Levels		
	Low (−1)	Medium (0)	High (+1)
X1: P.O. amount (mg)	200	400	600
X2: Cholesterol amount (mg)	25	50	100
X3: Brij 30 amount (mg)	10	20	30
Responses (Dependent variables)	Goal		
Y1: P.S (nm)	Minimize		
Y2: PDI (nm)	Minimize		
Y3: Z.P (mV)	Maximize		
Y4: E.E (%)	Maximize		

**Abbreviations:** P.O., Pumpkin seed oil; P.S, Particle size; PDI, polydispersity index; Z.P, zeta potential, and E.E, entrapment efficiency.

**Table 2:** Experimental runs, composition, independent variables, and measured responses of the I-Optimal response surface design for the GNS-PKs formulations. Data are presented as mean ± SD (*n* = 3).

Run	Factors			Responses			
	A: P.O. Amount (mg)	B: Cholesterol Amount (mg)	C: Brij 30 Amount (mg)	P.S (nm)	PDI	Z.P (mV)	E.E (%)
**1**	**400**	**25**	**30**	**170 ± 0.98**	**0.27 ± 0.05**	**−42 ± 0.87**	**92 ± 0.67**
2	200	50	30	210 ± 0.34	0.21 ± 0.03	−40 ± 0.42	90 ± 0.34
3	600	100	30	444 ± 0.97	0.25 ± 0.02	−39 ± 0.76	88 ± 0.65
4	600	50	20	345 ± 0.67	0.49 ± 0.05	−40 ± 0.88	78 ± 0.67
5	200	25	20	342 ± 0.65	0.35 ± 0.02	−38 ± 0.45	68 ± 0.87
6	400	50	10	234 ± 0.56	0.44 ± 0.03	−40 ± 0.61	89 ± 0.34
7	400	100	10	345 ± 0.43	0.41 ± 0.01	−38 ± 0.56	67 ± 0.55
8	200	100	10	451 ± 0.51	0.36 ± 0.03	−35 ± 0.34	79 ± 0.67
9	600	50	20	389 ± 0.54	0.59 ± 0.01	−29 ± 0.75	66 ± 0.91
10	400	50	10	321 ± 0.71	0.34 ± 0.01	−33 ± 0.97	82 ± 0.65
11	600	100	10	345 ± 0.67	0.51 ± 0.03	−30 ± 0.54	64 ± 0.59
12	600	50	20	459 ± 0.75	0.41 ± 0.02	−28 ± 0.66	68 ± 0.16
13	600	25	30	343 ± 0.76	0.48 ± 0.05	−29 ± 0.46	69 ± 0.54
14	200	25	10	345 ± 0.87	0.46 ± 0.04	−43 ± 0.66	92 ± 0.23
15	200	100	30	399 ± 0.61	0.29 ± 0.01	−41 ± 0.89	87 ± 0.49
16	600	25	10	298 ± 0.54	0.52 ± 0.04	−28 ± 0.43	65 ± 0.19
17	400	100	20	501 ± 0.46	0.34 ± 0.02	−29 ± 0.76	66 ± 0.87
18	400	100	20	345 ± 0.65	0.35 ± 0.04	−39 ± 0.78	65 ± 0.68
19	400	50	10	285 ± 0.56	0.26 ± 0.01	−37 ± 0.91	79 ± 0.51
20	400	25	20	345 ± 0.76	0.44 ± 0.03	−36 ± 0.55	78 ± 0.56

**Table 3:** Output data of the factorial design and predicted and observed values for the optimized formula (R1).

Responses	P.S	PDI	Z.P	E.E%
**Minimum**	170	0.21	−28	64
**Maximum**	501	0.59	−43	92
**Model**	Quadratic	Linear	Linear	Quadratic
**F** **-value**	16.431	8.555	9.132	6.817
***p*** **-value**	0.0023	0.009	0.008	0.025
**R^2^**	0.806	0.496	0.383	0.767
**Adjusted R^2^**	0.632	0.4011	0.268	0.558
**Predicted R^2^**	0.458	0.2208	0.0476	0.194
**Adequate precision**	7.421	8.428	5.226	6.311
**Significant factors**	B	A, C	A	A, C

**Table 4:** Stability study for optimized Genistein-Punpkisomes (GNS-PKs) formulations (Run 1).

Storage Time	Refrigerated Temperature (4 ± 1 °C)			Ambient Temperature				
	PS (nm)	PDI (nm)	Z.P (mV)	E.E (%)	PS (nm)	PDI	Z.P (mV)	E.E (%)
After 24 h	171 ± 0.76	0.27 ± 0.02	−41 ± 0.87	92.00 ± 0.63	170 ± 0.98	0.27 ± 0.05	−42 ± 0.87	92 ± 0.67
3 months	187 ± 0.45	0.29 ± 0.01	−39 ± 0.76	89.00 ± 0.29	188 ± 0.49	0.31 ± 0.08	−38 ± 0.23	87.00 ± 0.93
6 months	193 ± 0.93	0.34 ± 0.15	−37 ± 0.0.78	85.01 ± 0.49	198 ± 0.98	0.48 ± 0.05	−35 ± 0.01	83.01 ± 0.99

Data displays mean ± SD of three independent tests (*n* = 3).

**Table 5:** Physical characteristics of multifunctional pullulan microneedle patches.

MNs	Length (µm)	Tip Diameter (µm)	Base Diameter (µm)	Drug Content (%)	Length After Forces Applied per Array (µm)		
					250 gm	500 gm	1000 gm
M1	285.0 ± 0.6	4.0 ± 0.8	65.0 ± 0.9	72.4 ± 1.5	250.0 ± 0.8	230.0 ± 0.9	205.0 ± 2.0
M2	295.0 ± 0.9	4.5 ± 0.7	76.0 ± 1.1	85.7 ± 1.2	265.0 ± 0.6	255.0 ± 0.5	220.0 ± 0.9
M3	298.0 ± 0.7	5.0 ± 0.6	85.0 ± 1.2	93.1 ± 0.9	285.0 ± 0.9	277.0 ± 0.8	265.0 ± 1.0

**Table 6:** Mathematical models and their correlation coefficients for different formulations of Genistein.

Parameter	Free GNS	GNS-PKs	GNS-PKs MNs
**Q24% (percent released at 24 h)**	100%	60%	49%
**Jss (µg/cm^2^/h)**	(no steady state, burst release)	0.208	0.250
**Zero-order R^2^**	0.928	0.684	0.799
**First-order R^2^**	0.869	0.585	0.662
**Higuchi R^2^**	0.982	0.879	0.941
**Best-fit model**	Higuchi	Higuchi	Higuchi
**Mechanism**	Fickian diffusion	Fickian diffusion from pumpkisomes	Fickian diffusion through MNs, Pullulan, and pumpkisomes

**Table 7:** Effects of different formulations of genistein on the body weight of female Swiss albino mice.

Groups	Initial Body Weight (g)	Body Weight After Treatment
Group I	25 ± 0.12	52 ± 0.23
Group II	24± 0.19	26 ± 0.19
Group III	25± 0.23	35 ± 0.32
Group IV	25 ± 0.12	40± 0.15
Group V	23 ± 0.23	49 ± 0.29

**Note**: Group I (normal group, negative control); Group II (disease model group, positive control); Group III (Ehrlich + treated with GNS loaded with gel containing 2% (*w*/*w*) HPMC); Group IV (Ehrlich + treated with GNS-PKs loaded with gel containing 2% (*w*/*w*) HPMC); Group V (Ehrlich + GNS-PKs-loaded Pullulan MNs).

**Table 8:** Effects of different formulations of genistein on total bilirubin, serum glutamic oxaloacetic transaminase (SGOT), serum glutamate pyruvate transaminase (SGPT), alkaline phosphatase (ALP), total cholesterol, triglycerides, high-density lipoprotein (HDL) cholesterol, urea, uric acid, creatinine, estrogen, and progesterone hormone levels in female Swiss albino mice.

Parameter	GI	GII	GIII	GIV	GV
Total Bilirubin (mg/dL)	5.43 ± 0.15	60.43 ± 0.32 ****	30.32 ± 0.99 ***	12.43 ± 0.45 **	7.43 ± 0.34 ^ns^
SGOT (μ/L)	44 ± 0.54	145 ± 0.09 ****	86 ± 0.99 ***	67 ± 0.98 **	54 ± 0.12 ^ns^
SGPT (μ/L)	54 ± 0.98	125 ± 1.23 ****	110 ± 0.98 ***	89 ± 0.32 **	67 ± 0.87 ^ns^
ALP (μ/L)	150 ± 0.67	430 ± 1.39 ****	369 ± 0.94 ***	239 ± 0.68 **	180 ± 1.65 *
Total Cholesterol (mg/dL)	178 ± 1.14	270 ± 1.31 ****	232 ± 0.38 **	201 ± 1.43 *	186 ± 0.56 ^ns^
Triglycerides (mg/dL)	89 ± 0.54	210 ± 0.45 ****	198 ± 0.33 ***	157 ± 1.32 **	137 ± 0.66 ***
HDL Cholesterol (mg/dL)	89 ± 0.09	24 ± 0.78 ****	29 ± 0.84 ***	49 ± 0.99 **	76 ± 0.45 ***
Urea (mg/dL)	38 ± 0.23	115 ± 0.87 ****	89 ± 0.59 ***	67 ± 0.21 **	43 ± 0.32 *
Uric acid (mg/dL)	5 ± 0.99	65 ± 0.13 ****	49 ± 0.34 ***	29 ± 0.54 **	7.41 ± 0.14 *
Creatinine (mg/dL)	0.68 ± 0.81	40 ± 0.39 ****	34 ± 0.68 ****	11.23 ± 0.97 ***	6.43 ± 0.99 **
Progesterone.H (ng/mL)	61.21 ± 0.39	90.32 ± 0.92 ****	83.21 ± 0.95 *	69.43 ± 0.22 ^ns^	64 ± 0.13 ^ns^
Estrogen.H (Pg/mL)	66.8 ± 0.58	120 ± 0.78 ****	99.11 ± 0.45 **	86 ± 0.34 *	72 ± 0.54 ^ns^

**Note**: Group I (normal group, negative control); Group II (disease model group, positive control); Group III (Ehrlich + treated with GNS loaded with gel containing 2% (*w*/*w*) HPMC); Group IV (Ehrlich + treated with GNS-PKs loaded with gel containing 2% (*w*/*w*) HPMC); Group V (Ehrlich + GNS-PKs -loaded Pullulan MNs). Data are expressed as mean ± SD (*n* = 10). * *p* < 0.05, ** *p* < 0.01, *** *p* < 0.001, **** *p* < 0.0001, and ns (not significant) versus the negative control.

## Data Availability

The data presented in this study are available on request from the corresponding authors.

## Abbreviations

The following abbreviations are used in this manuscript:

**Abbreviation**	**Definition**
BC	Breast Cancer
SEC	Solid Ehrlich Carcinoma
EAC	Ehrlich Ascites Carcinoma
GNS	Genistein
PKs	Pumpkisomes
GNS-PKs	Genistein-loaded Pumpkisomes
MNs	Microneedles
GNS-PKs/MNs	Genistein-loaded Pumpkisomes in Microneedles
P.O.	Pumpkin Seed Oil
EE%	Entrapment Efficiency Percentage
PS	Particle Size
PDI	Polydispersity Index
Z.P	Zeta Potential
HPMC	Hydroxypropyl Methylcellulose
PU	Pullulan
CMC	Carboxymethyl Cellulose
HA	Hyaluronic Acid
SEM	Scanning Electron Microscopy
TEM	Transmission Electron Microscopy
FTIR	Fourier Transform Infrared Spectroscopy
DSC	Differential Scanning Calorimetry
HPLC	High-Performance Liquid Chromatography
PBS	Phosphate-Buffered Saline
IC_50_	Half-maximal Inhibitory Concentration
MCF-7	Human Breast Adenocarcinoma Cell Line
ELISA	Enzyme-Linked Immunosorbent Assay
EGFR	Epidermal Growth Factor Receptor
HER2	Human Epidermal Growth Factor Receptor 2
IL-6	Interleukin 6
COX-2	Cyclooxygenase 2
NF-κB	Nuclear Factor kappa-light-chain-enhancer of activated B cells
SOD	Superoxide Dismutase
CAT	Catalase
GSH	Glutathione
AST	Aspartate Aminotransferase
ALT	Alanine Aminotransferase
ALP	Alkaline Phosphatase
TG	Triglycerides
TC	Total Cholesterol Assay
